# Glycomics Microarrays Reveal Differential In Situ Presentation of the Biofilm Polysaccharide Poly-*N*-acetylglucosamine on *Acinetobacter baumannii* and *Staphylococcus aureus* Cell Surfaces

**DOI:** 10.3390/ijms21072465

**Published:** 2020-04-02

**Authors:** Andrea Flannery, Marie Le Berre, Gerald B. Pier, James P. O’Gara, Michelle Kilcoyne

**Affiliations:** 1 Carbohydrate Signalling Group, Discipline of Microbiology, National University of Ireland Galway, H91 TK33 Galway, Ireland; aaandreaf@gmail.com; 2 Infectious Disease Laboratory, Discipline of Microbiology, National University of Ireland Galway, H91 TK33 Galway, Ireland; jamesp.ogara@nuigalway.ie; 3 Advanced Glycoscience Research Cluster, School of Natural Sciences, National University of Ireland Galway, H91 TK33 Galway, Ireland; marie.leberre@nuigalway.ie; 4 Division of Infectious Diseases, Department of Medicine, Brigham and Women’s Hospital, Harvard Medical School, Boston, MA 02115, USA; gpier@bwh.harvard.edu

**Keywords:** biofilm, glycomics microarrays, bacterial adhesins, polysaccharide, *Staphylococcus aureus*, *Acinetobacter baumannii*, poly-*N*-acetylglucosamine, PNAG, lectin

## Abstract

The biofilm component poly-*N*-acetylglucosamine (PNAG) is an important virulence determinant in medical-device-related infections caused by ESKAPE group pathogens including Gram-positive *Staphylococcus aureus* and Gram-negative *Acinetobacter baumannii*. PNAG presentation on bacterial cell surfaces and its accessibility for host interactions are not fully understood. We employed a lectin microarray to examine PNAG surface presentation and interactions on methicillin-sensitive (MSSA) and methicillin-resistant *S. aureus* (MRSA) and a clinical *A. baumannii* isolate. Purified PNAG bound to wheatgerm agglutinin (WGA) and succinylated WGA (sWGA) lectins only. PNAG was the main accessible surface component on MSSA but was relatively inaccessible on the *A. baumannii* surface, where it modulated the presentation of other surface molecules. Carbohydrate microarrays demonstrated similar specificities of *S. aureus* and *A. baumannii* for their most intensely binding carbohydrates, including 3′ and 6′sialyllactose, but differences in moderately binding ligands, including blood groups A and B. An *N*-acetylglucosamine-binding lectin function which binds to PNAG identified on the *A. baumannii* cell surface may contribute to biofilm structure and PNAG surface presentation on *A. baumannii*. Overall, these data indicated differences in PNAG presentation and accessibility for interactions on Gram-positive and Gram-negative cell surfaces which may play an important role in biofilm-mediated pathogenesis.

## 1. Introduction

**Figure 1:**
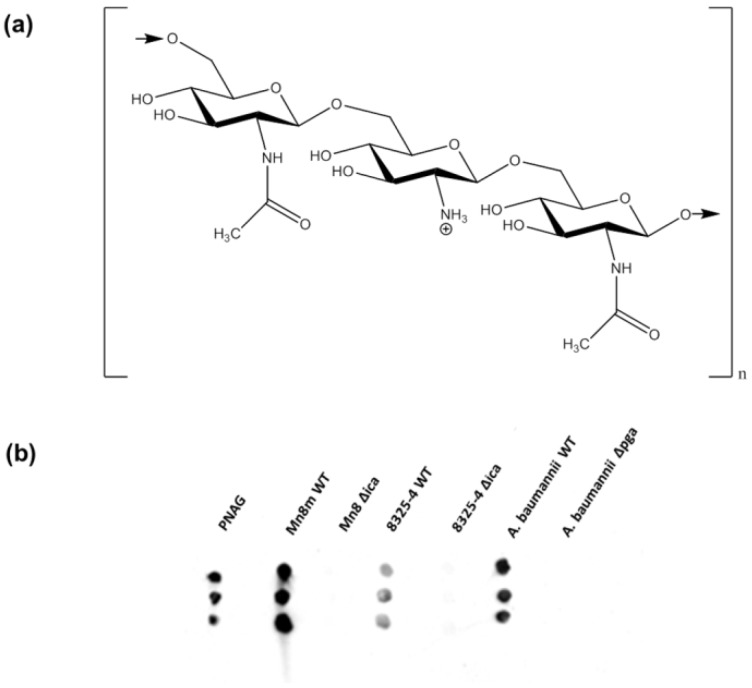
Structure of PNAG and verification of PNAG production. (**a**) Structure of partially deacetylated PNAG. Modifications of PNAG such as deacetylation and *O*-succinylation vary depending on bacterial genus and strain [32]. (**b**) Dot blot of heat-killed *S. aureus* Mn8m, *S. aureus* 8325-4 and *A. baumannii* S1 WT and mutant strains cultured under PNAG-promoting conditions detected by anti-PNAG mAb. The same cell numbers were loaded for comparison between strains (approximately 2 × 10^6^ cells).

Biofilms are formed by bacteria to adapt to environmental changes and protect themselves from the host immune system and other environments. Composed of microbial cells, exopolysaccharides, extracellular DNA (eDNA) and proteins, biofilms account for over 80% of microbial infections in humans [1] and are a major cause of hospital-acquired infections, notably associated with medical device infections [2]. In Europe, these infections resulted in 16 million extra days in hospital between 1995 and 2010, costing €7 billion and 37,000 deaths, while in the USA in the same period, 1.7 million patients acquired an infection in hospitals and 99,000 died from these infections [3]. Gram-positive *Staphylococcus aureus* and Gram-negative *Acinetobacter baumannii* are leading causes of hospital-acquired biofilm infections and members of the antibiotic-resistant ‘ESKAPE’ group of pathogens [4]. Both *S. aureus* and *A. baumannii* produce poly-*N*-acetylglucosamine (PNAG, Figure 1a) as a major component of their biofilm matrix as well as retaining PNAG on their cell surfaces. PNAG plays a fundamental role in the adhesion of *S. aureus* and *A. baumannii* cells within the biofilm matrix and has been implicated as a virulence factor important for *S. aureus* pathogenesis [5,6]. In contrast, there has been no correlation between PNAG production and *A. baumannii* virulence to date [7]. Correlations have been made between *S. aureus* antibiotic susceptibility and PNAG production, and between antibiotic susceptibility and biofilm formation in *A. baumannii* [7,8,9]. However, the presentation of PNAG in situ on the bacterial cell surface, PNAG interaction(s) and recognition by the host’s innate immune system, and the consequent effects on the immune system are still uncertain [5,10]. A better understanding of the presentation and accessibility of this important biofilm component on the bacterial cell surface could help to shed light on host–pathogen interactions and immune evasion.

Biofilms can be regarded as dynamic and responsive to the environment, and PNAG expression is influenced by a range of environmental factors including the availability of glucose, urea, and ethanol [11,12,13]. To our knowledge, it is not known whether altered growth conditions cause alterations in PNAG presentation on the bacterial surface or variations in the interactions of PNAG, although differences in surface glycosylation have been noted by lectin agglutination assays for methicillin-resistant *S. aureus* (MRSA) clinical strains under different culture conditions [14]. It is challenging to characterise and analyse biofilm and biofilm components, and laser microscopy in combination with fluorescently labelled lectins is one of the most common methods currently used to characterise biofilm carbohydrate and glycoconjugate content [15]. However, this method does not lend itself well to high throughput or multi-omics strategies, in particular glycomics, which is important for understanding host–pathogen interactions [16]. 

In addition to exopolysaccharides, secreted extracellular proteins, cell surface adhesins and protein subunits of flagella and pili participate in biofilm assembly and some of these bacterial proteins have a lectin function [17]. Recently, the outer-membrane-bound *P. aeruginosa* lectin, LecB (or PA-IIL), was shown to bind to the secreted biofilm exopolysaccharide Psl and thereby tether the bacterium to the biofilm matrix and facilitate biofilm assembly [18]. To date, only limited carbohydrate binding specificities have been characterised for *S. aureus* or *A. baumannii*, but to the best of our knowledge, there has been no investigation of the role of bacterial surface lectins in *S. aureus* or *A. baumannii* binding to biofilm component polysaccharides or in biofilm assembly or presentation. 

Lectin microarrays have been used for profiling bacterial surface glycosylation [19,20,21], while carbohydrate microarrays have been used to characterise the structural specificity of bacterial interactions [22]. However, glycomic microarrays have not been used to examine the presentation or interactions of intact biofilm components in situ on the bacterial cell surface, bacterial surface lectins potentially involved in biofilm assembly, or any potential influence of biofilm components on the presentation of other bacterial surface molecules. In this work, we describe the use of a lectin microarray to examine the in-situ presentation of PNAG on the cell surface of methicillin-sensitive *S. aureus* (MSSA) and MRSA strains and a clinical isolate of *A. baumannii*. The plant lectins wheatgerm agglutinin (WGA) and succinylated WGA (sWGA) were the only lectins to bind to PNAG alone from a panel of 48 lectins assessed. We observed differential surface presentation of PNAG between the Gram-positive and Gram-negative species, and differences in accessibility of PNAG for interactions with lectins. Further, carbohydrate microarrays identified the carbohydrate binding specificity of all strains in this study and revealed the binding of a surface lectin function to PNAG for *A. baumannii*. This approach contributes to the glycomics of multi-omics strategies for understanding host–pathogen interactions [16] and could provide further insights into biofilm-mediated pathogensis.

## 2. Results

### 2.1. Bacterial Strain Selections and Verification of Biofilm Production

The MSSA strains 8325-4 and Mn8m were selected as Gram-positive organisms that produce PNAG-predominant biofilm (Table 1 and Table S1) [23,24]. In *S. aureus*, PNAG is produced by proteins encoded in the *ica* operon and thus the ∆*ica* mutants of the *S. aureus* strains [25,26] were included in this study. The MRSA clinical isolate strain BH1CC has an *ica* operon but does not produce PNAG. Instead, eDNA is the main biofilm component [27]. *S. aureus* BH1CC wild type (WT) and the ∆*ica* mutant were included for comparison with the MSSA strains. In some species of Gram-negative bacteria, PNAG is synthesised by proteins produced by the *pga* operon, so the PNAG-producing clinical isolate *A. baumannii* strain S1 wild-type (WT) and its ∆*pga* mutant [28] were also included. Anti-PNAG monoclonal antibody (mAb) confirmed that the PNAG-producing strains *S. aureus* 8325-4, *S. aureus* Mn8m and *A. baumannii* S1, cultured under the biofilm-promoting conditions of media supplemented with 1% glucose or 4% NaCl, retained PNAG in situ on the cell surface under experimental conditions, while the ∆*ica* and ∆*pga* mutants did not produce any PNAG as expected (Figure 1b).

Crystal violet biofilm assays confirmed that *S. aureus* strains 8325-4 and Mn8m WT had increased biofilm formation in the presence of 1% glucose and/or 4% NaCl (Figure S1), and that this biofilm was primarily composed of PNAG (Figure S1), in agreement with previous reports [9,25,29,30,31]. *S. aureus* BH1CC WT had increased biofilm formation in the presence of 1% glucose and decreased or abolished biofilm in 4% NaCl (Figure S1), in agreement with previous reports [8,26,27]. PNAG was not involved in *S. aureus* BH1CC biofilm formation, as expected, and PNAG contributed to *A. baumannii* S1 biofilm formation in the presence of 1% glucose, as expected [28] (Table 1 and Figure S1).

### 2.2. Lectin Recognition of PNAG and Carbohydrate-mediated Binding Inhibition

WGA, which has binding specificity for both *N*-acetylglucosamine (GlcNAc) and sialic acid residues (Table S2), has been used as a ‘gold standard’ to detect and indicate the presence of PNAG within a biofilm matrix and on bacterial cell surfaces [33,34,35]. However, WGA does not exclusively recognise PNAG but also binds to other GlcNAc-containing bacterial cell surface molecules such as peptidoglycan [36,37,38]. As removal of PNAG in the Δ*ica*/Δ*pga* mutant strains uncovers or exposes other abundant and prominent GlcNAc-containing structures such as peptidoglycan on the bacterial cell surface, which in turn then become the main contributors to lectin binding interactions, simply comparing the different binding interactions of ∆*ica* or ∆*pga* mutants to WT alone will not serve to determine which lectins have preferential (but not necessarily exclusive) binding to PNAG. Therefore, to initially clarify lectin binding to PNAG alone, PNAG was purified from *S. aureus* Mn8m culture by ethanol precipitation, enzymatic digestion and size exclusion chromatography. Modifications of PNAG such as deacetylation and *O*-succinylation vary depending on bacterial genus and strain [32]. ^1^H NMR spectroscopy confirmed the purity and identity of the PNAG preparation which was approximately 5% *N*-deacetylated (Figure S2) and not *O*-succinylated, consistent with the previous report [39]. Lipoteichoic acid (LTA) and peptidoglycan are major cell wall components of Gram-positive bacteria and their structure varies between species [40]. Antibody dot blots demonstrated the presence of a trace amount of LTA (0.35% (*w/w*)) in the PNAG preparation and confirmed that peptidoglycan was not present (Figure S3). 

**Figure 2:**
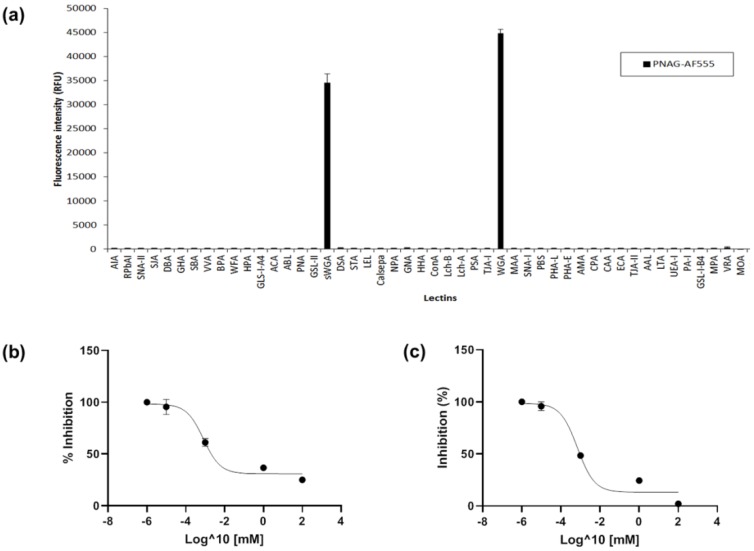
Lectin recognition of PNAG alone. (**a**) Lectin microarray profile of fluorescently labelled PNAG purified from *S. aureus* Mn8m culture. Bars represent the binding intensity of the mean of three experiments with error bars of +/−1 standard deviation (SD) of the mean. (**b**) Nonlinear fit transformation of GlcNAc inhibition PNAG binding to sWGA intensity data. Data points are the mean of three experiments with error bars of +/−1 SD of the mean. (**c**) Nonlinear fit transformation of GlcNAc inhibition of PNAG binding to WGA intensity data. Data points are the mean of three experiments with error bars of +/−1 SD of the mean.

The PNAG preparation was fluorescently labelled utilising its free amine groups (Figure 1a) and incubated on the lectin microarray. PNAG bound to only two lectins, sWGA, which binds to GlcNAc only (Table S2), and WGA (Figure 2a), and displayed a slightly higher binding intensity to WGA compared to sWGA. In agreement with these data, WGA and sWGA were previously demonstrated to bind to *S. epidermidis* ‘slime’, of which the major component is PNAG, using lectin histochemistry and transmission electron microscopy for detection [34]. The trace of contaminating LTA could potentially have contributed to lectin binding. However, since *S. aureus* Mn8m LTA has a diglucosyl (Glc-β-(1,6)-Glc) unit and does not contain any GlcNAc [41,42], it is therefore unlikely that LTA contributed to the GlcNAc-binding lectins observed here.

Although plant lectins are often used as tools to distinguish microbes, bacterial glycosylation is quite different to mammalian glycosylation for which plant lectin specificities have been mainly characterised, and carbohydrate-mediated binding for bacterial molecules should be confirmed [20]. Free GlcNAc inhibited selective PNAG binding to sWGA and WGA (Figure S4), while free mannose (Man) did not inhibit PNAG binding to sWGA and WGA as expected, confirming the specific carbohydrate-mediated binding of PNAG to the lectins. The half maximal inhibitory concentration (IC_50_) is a measure of how effective a sugar is for inhibiting lectin binding, and in this context, a lower IC_50_ value indicates that less GlcNAc was needed to compete for lectin binding to PNAG. The IC_50_ value generated by GlcNAc inhibition of PNAG binding to sWGA was 0.8161 μM (R^2^ = 0.9732) and to WGA was 0.6995 μM (R^2^ = 0.9641) (Figure 2b,c).

### 2.3. PNAG Presentation and Accessibility In Situ on Bacterial Cell Surface

To clarify the contribution and accessibility of cell-surface-retained PNAG to lectin binding of the whole bacteria, the surface glycosylation of the mutant strains was compared to that of the WTs cultured under the biofilm-promoting condition which produced most biofilm, which was supplemented with glucose for *S. aureus* strains BH1CC and Mn8m and *A. baumannii* and with NaCl for the *S. aureus* strain 8325-4 (Figure S1). All bacterial strains were initially titrated for optimal dye concentration for staining and cell number for incubation on lectin microarrays (Supplementary Materials and Figures S5–S8). As *S. aureus* BH1CC had the lowest fluorescence following staining compared to the other two *S. aureus* strains and *A. baumannii*, an optimal cell dilution of 50 µL was selected from the *S. aureus* BH1CC lectin microarray titration (Figure S8) for consistency across strains. 

**Figure 3:**
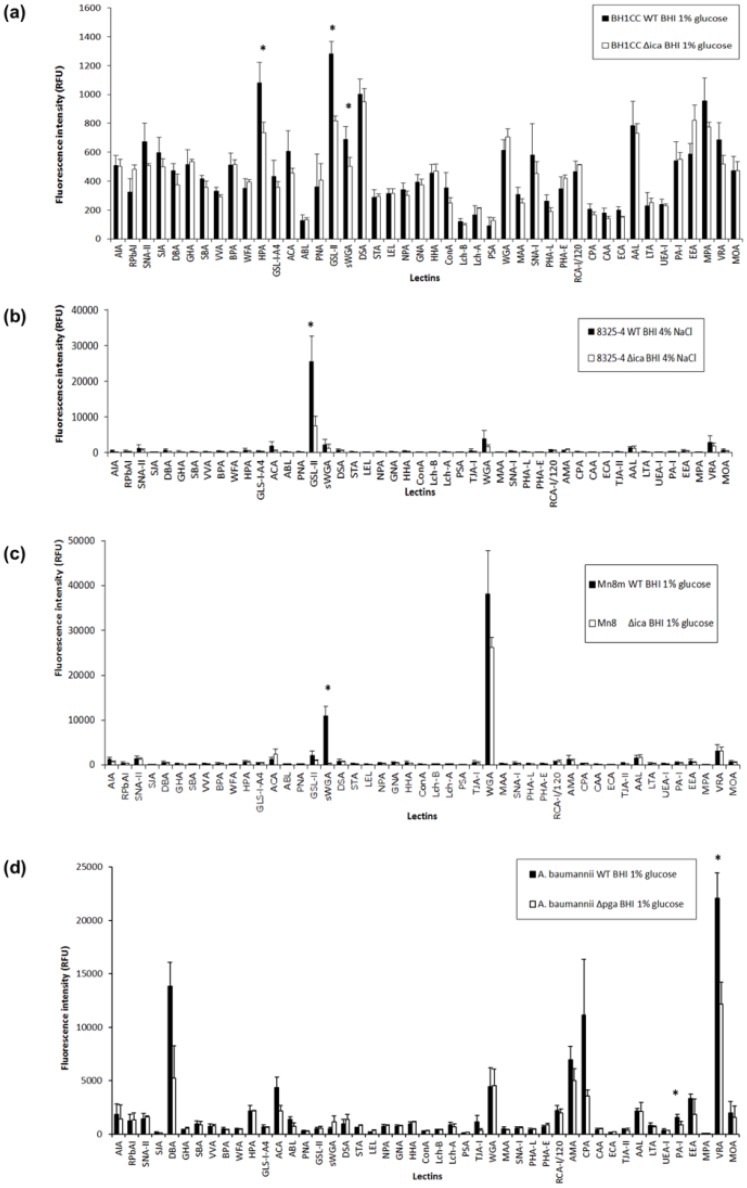
Surface glycosylation profiles of WT bacterial strains grown in BHI media supplemented with glucose or NaCl. Bar charts represent binding intensities of bacteria to lectins on the lectin microarray. (**a**) *S. aureus* BH1CC WT and ∆*ica* mutant bacterial strains grown in BHI media with 1% glucose, (**b**) *S. aureus* 8325-4 WT and ∆*ica* mutant bacterial strains grown in BHI media with 4% NaCl, (**c**) *S. aureus* Mn8m and Mn8 ∆*ica* mutant bacterial strains grown in BHI media with 1% glucose, and (**d**) *A. baumannii* WT and ∆*pga* grown in BHI media with 1% glucose. Bars represent the mean of three experiments with error bars of +/−1 SD of the mean. * represents significant difference (*p* ≤ 0.05, calculated by Student’s t-test, two tailed) in binding between WT and ∆*pga*/ ∆*ica*.

In MRSA clinical isolates, glucose promotes biofilm formation via an *ica*-independent mechanism that involves extracellular surface proteins, such as FnBPAB and eDNA [8,26,43]. MRSA strain BH1CC WT and Δ*ica* mutant cultured in BHI glucose exhibited overall very low binding intensities (<1500 RFU) on the lectin microarray by comparison to the MSSA strains and *A. baumannii* (Figure 3). This may have been due to the relatively thicker cell wall of MRSA strains compared to the MSSA strains which did not facilitate as much dye entering the cell for staining [44,45], or because this strain had a more efficient efflux pump and thus did not retain as much dye as the other MSSA and *A. baumannii* strains. It may also indicate that the MRSA strain BH1CC cell surface glycosylation is not very accessible or prominent in this format, in addition to the overall lower dye incorporation. *S. aureus* BH1CC WT bound with greatest relative intensity to *Helix pomatia* agglutinin (HPA), which has specificity for α-linked GalNAc residues, the GlcNAc-specific lectins *Griffonia simplicifolia* lectin-II (GSL-II) and *Datura stramonium* agglutinin (DSA), *Aleuria aurantia* lectin (AAL), which has specificity for α-(1,6)- and α-(1,3)-linked Fuc residues, and *Maclura pomifera* agglutinin (MPA), which has specificity for terminal α-linked Gal residues, but only moderate relative binding intensity was observed with sWGA and WGA (Table S2 and Figure 3a). While bacterial carbohydrate binding may not correspond exactly with the specificities for lectin established based on mammalian-type glycosylation, it is clear that these data indicated that PNAG was not the main contributor to *S. aureus* BH1CC WT cell surface or biofilm glycosylation, in agreement with the established absence of PNAG on the MRSA cell surface [8]. Additionally, the Δ*ica* mutant demonstrated slightly decreased binding to HPA, sWGA and GSL-II (Figure 3a). Although these changes in binding intensity were statistically significant, they were very slight in absolute intensity (<500 RFU) and thus likely indicated only very minor changes to surface glycosylation in the MRSA BH1CC Δ*ica* mutant compared to WT. 

When cultured supplemented in 4% NaCl, *S. aureus* 8325-4 WT bound with greatest intensity to the GlcNAc-specific lectins GSL-II, WGA, sWGA, and *Vigna radiata* agglutinin (VRA), which has specificity for terminal α-linked Gal residues (Table S2), with highest binding to GSL-II (Figure 3b). There was an overall trend of decreased binding of *S. aureus* 8325-4 ∆*ica* to all lectins compared to the WT, but only the decreased binding to GSL-II was significant (Figure 3b). It is unlikely that GSL-II binding to *S. aureus* 8325-4 WT was due to PNAG alone, as PNAG alone did not bind to GSL-II (Figure 2a). Instead, the decreased binding of the ∆*ica* mutant to GSL-II was likely due to GSL-II binding to another GlcNAc-containing surface molecule such as peptidoglycan or teichoic acid, binding which was augmented by the presence of PNAG in the WT. This kind of altered or modulated binding due to ‘neighbouring effects’ has been previously reported for anti-stage-specific embryonic antigen 3 (SSEA3, Gb5) antibody interactions [46]. From these data, it is clear that PNAG and other cell surface molecules were accessible and available for binding interactions, and PNAG did not completely obscure the cell surface from molecular interactions, even under biofilm-producing conditions. Alternatively, additional cell surface molecules may be secreted into the biofilm matrix and presented for binding along with the PNAG. The suggestion of incomplete surface coverage of *S. aureus* 8325-4 by PNAG is further supported by previous reports featuring electron microscopy images of *Staphylococcus* species in a biofilm matrix, which showed incomplete bacterial surface coverage by biofilm components [10,34,47]. 

On the other hand, *S. aureus* Mn8m produced approximately three times more PNAG than the 8325-4 strain (205% more PNAG compared to *S. aureus* 8325-4 by densitometry, Figure 1b), so PNAG may more fully enclose the strain Mn8m bacterial cell surface under biofilm-promoting conditions. *S. aureus* Mn8m WT bound with greatest intensity to the lectins GSL-II, sWGA, WGA, and VRA, with WGA binding of greatest relative intensity (Figure 3c). Binding to WGA and GSL-II was decreased for the *S. aureus* Mn8 ∆*ica* mutant compared to Mn8m, and sWGA binding was entirely absent for the mutant (Figure 3c). Thus, sWGA binding of the MSSA Mn8m WT was entirely due to PNAG alone, and no exposed surface molecules of the WT interacted with sWGA in addition to PNAG, supporting the proposal of more complete obscuring of the *S. aureus* Mn8m WT cell surface compared to *S. aureus* 8325-4 WT. Therefore, depending on surface coverage, PNAG may not be the most prominent molecule contributing binding interactions and/or it can serve to modulate binding of recognition molecules to cell surface components. 

*A. baumannii* S1 WT grown in biofilm-inducing glucose demonstrated binding to: *Dolichos biflorus* agglutinin (DBA), which has specificity for GalNAc residues; *Amaranthus caudatus* agglutinin (ACA), specific for sialylation and Gal-β-(1,3)-GalNAc; WGA; *Arum maculatum* agglutinin (AMA), specific for Gal-β-(1,4)-GlcNAc (*N*-acetyllactosamine (LacNAc)); *Cicer arietinum* agglutinin (CPA), specific for complex oligosaccharides; and VRA. Of these, the most intense binding was to DBA, CPA and VRA (Table S2 and Figure 3d). If lectin specificities for bacterial glycosylation are as previously characterised, this could indicate that *A. baumannii* has a cell surface rich in Gal and GalNAc-containing structures. Given the relatively lower binding to WGA and sWGA, PNAG was not the most prominent cell-surface-presented molecule for *A. baumannii*, and it was relatively less accessible for interactions compared to MSSA strains under biofilm-promoting conditions.

*A. baumannii* ∆*pga* demonstrated reduced binding to DBA, ACA, CPA and VRA in comparison to the WT, but not to WGA, sWGA or any other GlcNAc-specific lectins (Figure 3d). The lack of reduction in binding to GlcNAc-specific lectins for the mutant strain indicates that, even aside from relatively minor structural differences in terms of degree of deacetylation, PNAG was presented quite differently on the surface of *A. baumannii* compared to the MSSA strains. Although PNAG on both MSSA and *A. baumannii* surfaces was detectable by a recognition molecule specific for PNAG, the anti-PNAG mAb (Figure 1b), recognition molecules that are not specific for PNAG in particular, such as innate immune receptors, may not bind well to PNAG accessible in a different presentation, as demonstrated by the plant lectins here. Since DBA, ACA, CPA and VRA lectins mainly have specificity for Gal and GalNAc residues, these data indicate that PNAG on the surface of *A. baumannii* modulated the presentation of other cell surface components, such as lipopolysaccharides (LPS) or capsular polysaccharide (CPS). For example, CPS isolated from *A. baumannii* NIPH146 had a pentasaccharide repeating unit composed of Glc, Gal and GalNAc residues which contained a α-d-Gal*p*-(1,6)-β-d-Glc*p*-(1,3)-d-Gal*p*NAc trisaccharide fragment common among many *A. baumannii* strains [48], and the O-antigen isolated from *A. baumannii* strain 9 and ATCC 17961 LPSs consisted of Glc, GalNAc, Gal, GlcNAc and GlcNAc3NAcA residues [49]. Therefore, PNAG may have a role in modulating the interactions of other more prominent molecules of the *A. baumannii* surface with recognition molecule(s), rather than PNAG itself directly interacting with recognition molecules.

Taking all of the above into account, it is therefore not advisable to exclusively use WGA for identifying PNAG in biofilm or on cell surfaces. Instead, we suggest that sWGA may be a better indicator for the presence of PNAG in *S. aureus* cultures or biofilms, but a recognition molecule that is specific for the PNAG structure itself, such as mAb for PNAG, may be the best identification molecule across bacterial species. However, as demonstrated here, the use of the antibody alone cannot show conformational or presentation differences and it should be combined with a multi-interaction detection platform such as the lectin microarray for a more complete understanding.

Overall, these data demonstrate differences in surface presentation and accessibility of PNAG in situ on the bacterial cell surfaces, with complete surface coverage by PNAG indicated for MSSA Mn8m, incomplete PNAG surface coverage for MSSA 8325-4 with accessibility of other cell surface molecules for interactions, no PNAG on the MRSA cell surface as expected, and, although PNAG was present on *A. baumannii* cell surface, other surface molecules were the most accessible on *A. baumannii* and PNAG influenced their interactions.

### 2.4. Carbohydrate Specificity of A. baumannii and S. aureus Strains

We next investigated the carbohydrate binding specificities of *S. aureus* [50] and *A. baumannii* using carbohydrate microarrays (Tables S3 and S4). All WT and mutant strains were grown in biofilm-promoting conditions, as for lectin microarray profiling. Additionally, MRSA BH1CC was also cultured with NaCl supplementation, as salt promotes *icaA* transcription but does not promote biofilm formation in MRSA clinical isolates [8], and this condition would be useful to compare to the *ica*-independent mechanism promoted by inclusion of glucose that involves extracellular surface proteins, such as the fibronectin-binding protein (FnBP) FnBPAB, and eDNA [8,26,43]. For comparison, the MSSA strain 8325-4 was also grown in the presence of glucose, which also increases PNAG-mediated biofilm formation in this strain.

Overall, the most intensely binding ligands for MSSA strain 8325-4 WT cultured in BHI glucose were 3′-sialyllactose (3SLac, on neoglycoconjugate 3SLacHSA), 6′-sialyllactose (6SLac, on neoglyconjugate 6SLacHSA), H type II (H2, on neoglycoconjugate H2BSA), lacto-*N*-tetraose (LNT, on neoglycoconjugate LNTHSA), rhamnose (Rha, on neoglycoonjugate RhaBSA), α-linked mannose (Man, on neoglycoconjugate XManaBSA) and β-linked Glc (on neoglycoconjugate GlcbITCBSA) (Figure S10, Tables S3 and S4). When cultured in BHI NaCl, the binding pattern of *S. aureus* 8325-4 WT remained similar, with the most intense binding including the glycoprotein α-crystallin (a-C) and difucosyl-para-lacto-*N*-hexaose (DFPLNH, on the neoglycoconjugate DFPLNH) (Figure S11, Tables S3 and S4). The other MSSA strain Mn8m WT cultured in BHI glucose had a similar binding pattern to strain 8325-4 cultured under both conditions and bound most intensely to similar ligands, including a-C, 3SLac, 6SLac, H2, DFPLNH, LNT, Rha, α-linked Man and β-linked Glc, and additionally Lewis b (Leb, on neoglycoconjugate LebBSA) (Figure S12, Tables S3 and S4). 

**Figure 4:**
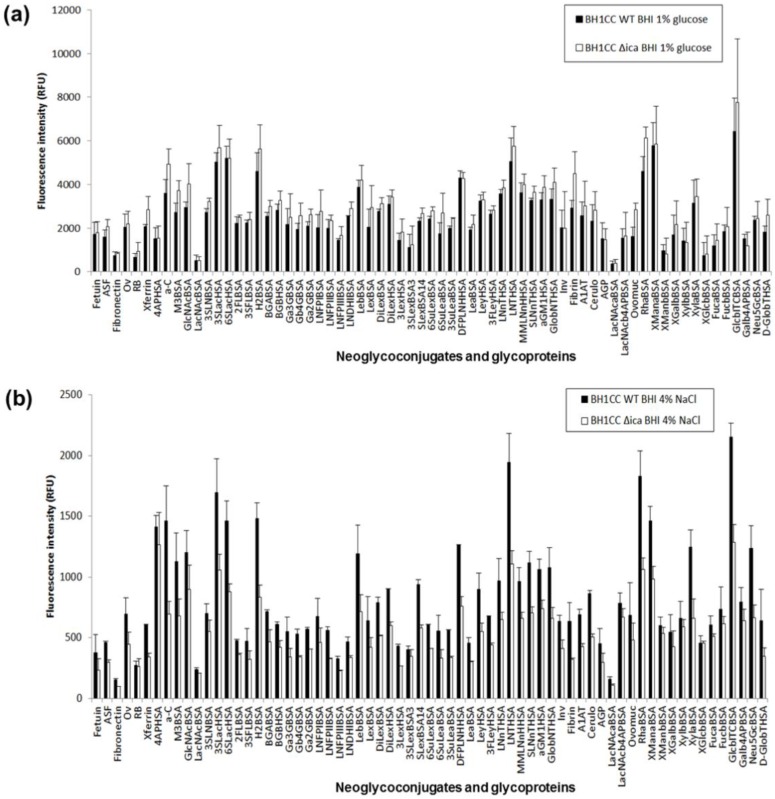
Carbohydrate microarray binding intensity profiles of *S. aureus* BH1CC WT and ∆*ica* grown in BHI supplemented with (**a**) 1% glucose and (**b**) 4% NaCl. Bars represent the mean of three experiments with error bars of +/−1 SD of the mean.

For the MRSA strain BH1CC cultured in BHI glucose, the overall binding intensities to carbohydrates were comparable in intensity to the MSSA strains, unlike the overall MRSA binding intensities for lectin microarrays despite the same staining. The most intense binding for *S. aureus* BH1CC cultured in BHI glucose was for ligands 3SLac, 6SLac, H2, DFPLNH, LNT, Rha, α-linked Man and β-linked Glc (Figure 4a, Tables S3 and S4). When cultured in BHI NaCl, the *S. aureus* strain BH1CC exhibited markedly lower overall intensity (<2500 RFU, Figure 4b) compared to culture in glucose, which may correspond to the much greater quantity of biofilm produced when *S. aureus* BH1CC is cultured in glucose compared to salt (Figure S1C). The most intensely binding ligands for this strain cultured with NaCl were the 4-aminophenyl linker (4AP, on the protein conjugate 4APHSA), a-C, 3SLac, 6SLac, H2, LNT, Rha, α-linked Man and β-linked Glc (Figure 4b). Although not among the most intensely binding ligands, under both culture conditions *S. aureus* BH1CC bound with moderate relative intensity to human matrix protein fibrinogen (fibrin, Tables S3 and S4) but very low relative intensity to fibronectin. Binding to fibrinogen was much greater in absolute intensity when cultured in glucose (approximately 3000 RFU, Figure 4a) compared to culturing in NaCl (approximately 200 RFU, Figure 4b). This may be due to the greater quantity of FnBPAB-mediated biofilm production under the glucose supplemented growth condition as expected (Table 1 and Figure S1c). Although FnBPAB is produced by *S. aureus* BH1CC (Table 1), it may not be among the most intensely binding ligands for this strain, as the major biofilm component is eDNA rather than FnBPs [27]. 

**Figure 5:**
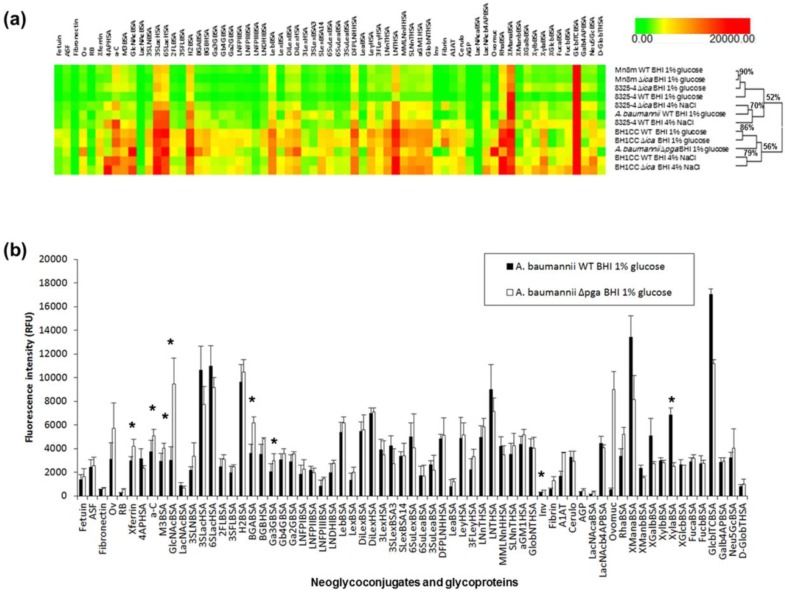
Carbohydrate binding intensities of bacterial strains. (**a**) Unsupervised hierarchical clustering of carbohydrate microarray binding intensities for *S. aureus* Mn8m, 8325-4, BH1CC and *A. baumannii* all grown in BHI supplemented with 1% glucose, and supplemented with 4% NaCl for *S. aureus* 8325-4 and BH1CC. Binding intensity data was scale-normalised to 20,000 RFU maximum and clustered using Hierarchical Clustering Explorer v3.0 with complete linkage and Euclidean distance. (**b**) Bar chart representing carbohydrate binding intensities of *A. baumannii* WT and ∆*pga* grown in BHI glucose. Bars represent the mean of three experiments with error bars of +/−1 SD of the mean. * represents significant difference (*p* ≤ 0.05, calculated by Student’s t-test, two tailed) in binding between WT and ∆*pga*.

Similarly to the *S. aureus* strains, *A. baumannii* WT cultured in BHI glucose bound most intensely to 3SLac, 6SLac, H2, LNTHSA, Rha, α-linked Man and β-linked Glc (Figure 5).

For all strains, the binding to Rha is unlikely to be biologically relevant to human infection, as Rha is a common component in plants and some bacteria (e.g., Mycobacterium) but does not occur in mammals, and indeed the linker may contribute to binding here. All strains also bound to β-linked Glc and α-linked Man, but only when the phenylisothiocyanate (ITC) linker was present. None of the strains bound with similar high intensity to β-linked Glc when it was presented with the 4AP linker (XGlcbBSA, Tables S3 and S4) nor to the α-linked Man presented as part of the Man-α-(1,3)-[Man-α-(1,6)-]Man trisaccharide structure (M3BSA) or in the high-mannose structures on the glycoprotein ribonuclease B (RB, Tables S3 and S4). The influence of the ITC linker on carbohydrate binding has been previously reported where Con A lectin, which normally has binding specificity for α-linked Man and Glc (Table S2), bound to Glc, which was β-linked with a phenylazo linker [51]. Thus, we hypothesize that the ITC linker is particularly accessible in these ITC-linked neoglycoconjugates and the interactions with bacteria were mediated, at least in part, by the ITC linker and not dependent on carbohydrate binding. 

Inhibition studies using free mono- or oligo-saccharides were not used here to verify carbohydrate-mediated bacterial binding, as many bacteria can ferment free carbohydrates and this can change the expression of surface molecules (e.g., glucose supplementation increases *S. aureus* biofilm expression as demonstrated in this work). Alternatively, structural specificity can be deduced and confirmed by including closely related structural variations or different presentations in the presented ligand panel, as demonstrated in the case above of the strains binding to α-linked Man on the ITC linker but not α-linked Man presented on the trisaccharide M3BSA or on RB, indicating that bacterial binding was not dependent on the carbohydrate in the case of neoglycoconjugate XManaBSA. 

All strains appeared to favour binding to 3′ and 6′ sialylated type II lactose (Lac, Gal-β-(1,4)-Glc), but not to 3′sialyl-*N*-acetyllactosamine (3′SLacNAc, Neu5Ac-α-(2,3)-Gal-β-(1,4)-GlcNAc, on the neoglycoconjugate 3SLNBSA, Table S3). Therefore, the *N*-acetylamino group on 3′SLacNAc may be inhibitory to binding. None of the strains favoured intense binding to unmodified LacNAc (Gal-β-(1,4)-GlcNAc, on the neoglycoconjugates LacNAcBSA, LacNAcaBSA and LacNAcb4APBSA, Tables S3 and S4) either. However, when LacNAc was substituted with terminal α-(1,2)-linked fucose (Fuc) in the H2 antigen (Fuc-α-(1,2)-Gal-β-(1,4)-GlcNAc), relatively intense binding occurred. This favoured binding to terminal α-(1,2)-linked Fuc was supported by moderate to intense binding of Leb (Fuc-α-(1,2)-Gal-β-(1,3)-[Fuc-α-(1,4)-]GlcNAc-β-(1,3)-Gal-β-(1,4)-Glc) by all strains. Interestingly, the Leb structure on a different neoglycoconjugate, LNDHIBSA, was not bound by the strains as intensely as LebBSA. This may be due to the higher substitution of Leb on the BSA backbone on LebBSA compared to LNDHIBSA (substitution of 10 compared to 7.5 (range 4–12), respectively), as even small differences ligand density can dramatically affect avidity of the interactions [52]. The different linkers of LebBSA and LNDHIBSA may also have a role in ligand presentation differences, but the linked effect may not have as much impact on the conformation or accessibility of longer oligosaccharides compared to monosaccharides.

The species and strains differed more in their moderate to low intensity binding. For example, all *S. aureus* WT strains in all culture conditions had low relative binding to Lewis x (Lex, on the neoglyconjugate LexBSA), blood group B (BGB, on neoglyconjugate BGBBSA) and blood group A (BGA, on neoglyconjugate BGABSA) (Figure 4 and Figures S9–S11), all of which do not have terminal α-(1,2)-linked Fuc, while *A. baumannii* WT also had low Lex binding but moderate binding to BGA and BGB (Figure 5b). Thus, these bacterial strains may have preferential binding to secretor hosts that have a functional *FUT2* gene, which makes the enzyme α-1,2-fucosyltransferase, rather than non-secretors who have a non-functional *FUT2* gene. Fucosylation plays an important role for host–microbe interactions, and secretor status is a genotypic factor that contributes to microbial diversity, particularly bifidobacteria diversity, in the intestinal microbiota [53]. Tissue specific expression of histo-blood group antigens indeed appears to influence *S. aureus* colonisation. Non-secretors of blood group O are more likely to carry *S. aureus* in their throat compared to BGA non-secretors, while secretors of blood group O appear to be protected [54]. The latter may be due to the production of mucus containing α-(1,2)-linked Fuc-expressing mucins in blood group O secretors constantly removing resident *S. aureus* from the throat with mucus turnover. In addition, although some strains of *S. aureus* have been shown to bind to Lewis a (Lea) via a specific adhesin [55], binding to Lea (on the neoglycoconjugate LeaBSA) was of relatively low intensity for all *S. aureus* strains and growth conditions assessed in this work, so Lea binding may be strain-specific. To the best of our knowledge, *A. baumannii* colonisation or infections have not been associated with histo-blood group antigens to date.

The WT MSSA strains cultured in glucose did not bind to either fibronectin or fibrinogen (relatively very low intensity binding, Figures S10 and S12), while *S. aureus* 8325-4 cultured with NaCl exhibited relatively low to moderate binding intensity (Figure S11) despite the known presence of FnBPs in *S. aureus*, which bind to both fibronectin and fibrinogen [56]. The relevant FnBP adhesins may not have been abundantly expressed without the presence of fibrinogen or fibronectin under these culture conditions. Alternatively, the *S. aureus* binding demonstrated in this work may reflect that *S. aureus* binding to the other presented ligands is actually relatively more intense or important than fibrinogen or fibronectin binding. Certainly, this is likely in the case of *S. aureus* BH1CC, where the majority biofilm component is eDNA and not FnBPAB [27].

### 2.5. Bacterial Lectin Function in A. baumannii Biofilm Assembly

Removal of PNAG may result in an overall increased or decreased carbohydrate binding of the mutants compared to WTs, but not result in a difference in the overall relative binding pattern. This may be due to reduced overall ‘stickiness’ of the bacterium with the loss of the PNAG or differences in the degree of dye uptake, which can affect the overall fluorescence of the bacteria and result in a ‘shelving effect’ despite loading the same cell number. Such a ‘shelving effect’ indicates an artefactual difference in overall binding intensity and is not representative of real binding differences between strains or conditions. To examine whether any carbohydrate binding was altered between the WT and Δ*ica* and Δ*pga* mutants and also mitigate against any bias introduced by potential artefactual differences, we first compared the scale-normalised carbohydrate binding profiles of all WT and mutant strains by hierarchical clustering (Figure 5a) to identify lectin functions that could play a role in biofilm assembly. Overall binding patterns that are different can then test individual binding interactions for statistical significance and for substantially increased or decreased binding differences (e.g., ≥50%) between the WT and mutant. This approach for identifying differences of potential biological consequence is particularly relevant to complex systems such as bacteria, which are more likely to have multiple surface lectins with different specificities and affinities that are expressed in different ratios under different conditions, rather than the much simpler ‘on–off’ or ‘binding–no binding’ approach, which is a more suitable approach for simple or single-component systems.

Two major groups were created by unsupervised hierarchical clustering, with one group containing both WT and ∆*ica* mutant MSSA strains, under all culture conditions, and the other group containing the MRSA strain WT and ∆*ica* mutant, under all culture conditions. The ∆*ica* mutants of *S. aureus* strains 8325-4 and Mn8m and the Mn8m WT when cultured in BHI glucose were essentially the same (90% similarity, Figure 5a), and also had high similarity to the *S. aureus* 8325-4 WT cultured in glucose, despite apparent differences of absolute intensity (rather than relative intensity) for strain 8325-4 (Figure S10). *S. aureus* 8325-4 WT and ∆*ica*, when cultured in BHI NaCl, were a little different in overall binding pattern compared to when cultured in BHI glucose (70% similarity, Figure 5a). Binding of the *S. aureus* 8325-4 ∆*ica* mutant cultured in BHI NaCl was significantly (*p* ≤ 0.05) increased compared to the WT to several structures: the H2 antigen (on the neoglycoconjugates H2BSA and 2FLBSA), Gal-β-(1,4)-Gal (on the neoglycoconjugate Gb4GBSA), Lewis y (Ley, on the neoglycoconjugate LeyHSA), monofucosyl monosialyllacto-*N*-neohexaose (on the neoglycoconjugate MMLNnHHSA) and α-linked Man (on the neoglycoconjugate XManaBSA, which is Man-α-ITC-BSA) (Figure S11). However, several of these significantly different binding interactions were not substantially different in magnitude. The only substantially increased binding (≥50%) of the ∆*ica* mutant compared to *S. aureus* 8325-4 WT was to fibronectin (139%), fibrinogen (102%), α-linked Man (on the neoglycoconjugate XManaBSA), β-linked Gal (87%, on the neoglycoconjugate XGalbBSA (Gal-β-ITC-BSA)) and β-linked Glc (61%, on the neoglycoconjugate GlcbITCBSA) (Figure S11). As discussed above in the previous section, the ITC linker may have a role in the substantially increased binding to the α-linked Man, β-linked Gal and β-linked Glc, as these large increases in binding were not observed for the same carbohydrates on different linkers or presentations. However, the altered binding to fibronectin and fibrinogen may indicate the increased importance of FnBP adhesins in biofilm assembly for *S. aureus* 8325-4 under these culture conditions.

Within the second group containing MRSA, the similarity of *S. aureus* BH1CC WT and ∆*ica* cultured in BHI glucose (86%, Figure 5a) showed that the binding pattern was essentially the same, with the same conclusion for *S. aureus* BH1CC WT and ∆*ica* cultured in BHI NaCl (79%, Figure 5a). However, the binding pattern differed for *S. aureus* BH1CC when cultured in NaCl compared to glucose supplementation. These differences were mainly in relative intensity of binding to the moderately bound ligands, including lower binding of the BHI NaCl culture to fibrinogen, 6-sulfoLewis x (6SuLex, on neoglycoconjugate SuLexBSA) and the 4AP linker. The overall similarity of carbohydrate binding pattern between the WT and mutants of the same *S. aureus* strains indicates that surface-bound or secreted adhesins with lectin function are not likely to have an important role in biofilm assembly or surface presentation for this species. In addition, these data demonstrate that different culture conditions impact on binding pattern, likely influencing the relative ratios of surface lectins produced rather than a simple ‘on-off’ expression.

*A. baumannii* WT and ∆*pga* mutant clustered separately into the two different major groups, with the WT clustering with the PNAG-producing MSSA strains and conditions and *A. baumannii* ∆*pga* clustering with the non-PNAG producing MRSA group (Figure 5a). *A. baumannii* ∆*pga* demonstrated significantly (*p* ≤ 0.05) increased binding in comparison to WT for the neoglycoproteins GlcNAcBSA, M3BSA, 3SLacHSA, Ga3GBSA, BGABSA, XylaBSA and GlcbITCBSA, and the glycoproteins ovalbumin (Ov), a-C, transferrin (Xferrin) and invertase (Inv) (Figure 5b, Tables S3 and S4). However, some of these binding differences were negligible in magnitude despite their statistical significance. Substantially increased binding (≥50%) for *A. baumannii* ∆*pga* was observed for the probes Ov (83%), GlcNAcBSA (212%), LexBSA (50%), RB (115%), 3SLNBSA (55%), BGABSA (71%), LNFPIIIBSA (71%), Inv (76%), human fibrinogen (116%), human alpha-1 antitrypsin (A1AT, 120%), LacNAcaBSA (128%), ovomucoid (ovomuc, 1522%), and RhaBSA (55%) (Figure 5b) in comparison to the WT, while GM1HSA (−135%) and XylaBSA (−63%) exhibited substantially decreased binding in comparison to the WT. 

The most substantial increase in binding of *A. baumannii* ∆*pga* was to ovomucoid from chicken egg white which has mainly tri- and penta-antennary complex-type N-linked glycans, with the most abundant structures having mainly terminal GlcNAc residues, and almost 80% of structures have bisecting GlcNAc [57]. Bacterial binding specificity for terminal β-linked GlcNAc residues was further supported by the substantially increased binding to GlcNAcBSA (also statistically significant) and A1AT. A1AT from healthy human plasma has complex-type N-linked glycosylation with mainly biantennary structures and some tri- and tetra-antennary. The majority of structures have terminal sialylation and galactosylation, but a proportion of the biantennary structures have terminal GlcNAc residues and bisecting GlcNAc. In addition, A1AT glycosylation has been shown to be altered with chronological age and to differ between males and females [58]. Thus, the *A. baumannii* ∆*pga* binding specificity for several carbohydrate structures was revealed, including terminal β-linked GlcNAc residues by the substantially increased binding to GlcNAcBSA, ovomucoid and A1AT compared to the WT.

The GlcNAc-binding lectin functionality on the surface of *A. baumannii* revealed by the removal of PNAG may have a functional role in binding PNAG tightly to the bacterial cell surface, making it less accessible for recognition molecules that are not specific for PNAG but influencing the presentation or accessibility of other cell surface molecules such as LPS. In *S. aureus*, PNAG is deacetylated by approximately 5% [39], imparting an overall positive charge. For biofilms that are dependent on extracellular surface proteins, such as those formed by MRSA, it has been proposed that eDNA acts as an electrostatic net that connects positively charged surface proteins in low pH environments within a biofilm matrix [59]. There are also many negatively charged molecules on the surface of bacteria, including teichoic acids, which impart an overall negative charge on the bacterial cell surface. Thus, it has been hypothesised that the positively charged amine groups on PNAG act as an electrostatic glue that interacts with these negative charges, helping to hold a biofilm matrix together [47] and thereby immobilising and presenting the PNAG on the *S. aureus* cell surface. However, *A. baumannii* PNAG is deacetylated by approximately 40% [28] and so has a correspondingly higher charge, while the Gram-negative bacterial cell surface is also typically negatively charged. Interestingly, it has been reported that Gram-negative strains that adhered to a positively charged surface ceased to grow, but there was no antimicrobial effect on Gram-positive bacteria [60]. Direct contact of this higher charged PNAG with the cell surface of the Gram-negative bacteria may be antimicrobial, so an ‘anchor’ or ‘buffer’ to keep the PNAG close to the surface but not touching it could be the required function fulfilled by the GlcNAc-binding surface lectin in *A. baumannii*. 

There are many bacterial surface-bound proteins with adhesin and lectin function involved in biofilm formation and organisation, including FnBPs and Protein A, which contains the GlcNAc-binding module LysM [61]. However no *S. aureus* lectins have been found to directly associate with PNAG to promote biofilm formation, in agreement with our data. Mutation of *lysM* in *A. baumannii* reduces biofilm formation [62], which supports a role for this GlcNAc-binding module for *A. baumannii* biofilm formation and thus LysM, may be a promising candidate for the *A. baumannii* GlcNAc-binding lectin identified in this work.

## 3. Discussion

**Figure 6:**
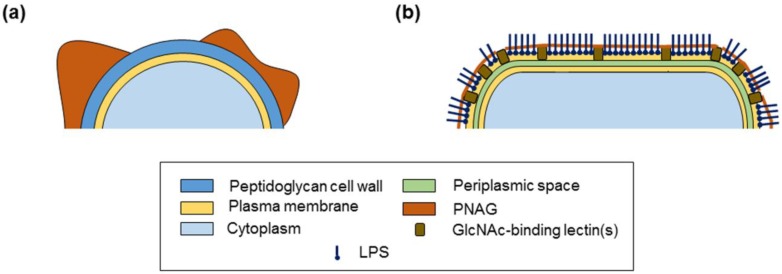
Model of proposed presentation of PNAG on the surface of (**a**) methicillin-sensistive *S. aureus*, and (**b**) *A. baumannii*.

The differential surface presentation of *A. baumannii* PNAG compared to *S. aureus* indicated by the carbohydrate binding data is in agreement with the different surface presentations and accessibility of PNAG between the species indicated by the lectin binding data. Based on the lectin microarray data, the surface molecules of MSSA strain 8325-4 were accessible and contributed to binding interactions, which suggested incomplete PNAG coverage of the bacterial cell surface (Figure 6a), similar to the partial surface coverage by biofilm previously indicated in scanning electron microscopy images of *S. epidermidis* with intact PNAG on the cell surface [10,63]. 

On the other hand, the highly deacetylated *A. baumannii* PNAG may support tight adherence to the cell surface, with the GlcNAc-binding lectin(s) occupied in anchoring the PNAG to the cell surface and providing a buffer to block direct contact between the highly charged polysaccharide and the cell surface. From our lectin microarray data, it is clear that PNAG is not the main surface molecule accessible by non-specific environmental recognition molecules, but it did influence the presentation and accessibility of the other surface molecules of *A. baumannii*, likely including LPS. Thus, we propose a model for PNAG presentation on the *A. baumannii* surface where PNAG is tightly adherent to the cell surface but not touching it and other surface molecules extend beyond the immobilised PNAG, with PNAG influencing their presentation (Figure 6b). 

The differentially presented PNAG on *A. baumannii* and *S. aureus* surfaces may help the PNAG perform different biological roles or physical functions for each species. For example, compared to *S. aureus*, PNAG on the surface of *A. baumannii* appears to play a role in biofilm integrity under shear force, whereas PNAG on MSSA plays a vital role in biofilm formation under static conditions [28]. Furthermore, the tightly adherent PNAG on the *A. baumannii* surface may contribute to its extraordinarily long survival time on abiotic surfaces under desiccated conditions, contributing to its persistence in clinical environments [64].

## 4. Materials and Methods 

### 4.1. Materials

Agar, Alexa Fluor^®^ 555 (AF555) carboxylic acid succinimidyl ester fluorescent label, Pierce™ enhanced chemiluminescence (ECL) Western blotting substrate, mouse anti-LTA IgG mAb, mouse anti-peptidoglycan IgG1 mAb (3F6B3 (10H6)), Nunc™ MicroWell™ (Nunclon (∆ surface) tissue culture-treated) 96-well microtitre plates and SYTO™ 82 nucleic acid stain were purchased from ThermoFisher Scientific (Dublin, Ireland). The Δ certification is a proprietary cell culture surface treatment that offers maximum adhesion for a broad range of cell types and is used for biofilm assays. Brain Heart Infusion (BHI) agar and crystal violet were obtained from Sigma-Aldrich Co. (Dublin, Ireland). Proteinase K was from QIAGEN (Hilden, Germany). Casein was purchased from BDH, Merck (Dublin, Ireland). Nexterion^®^ Slide H microarray slides were supplied by Schott AG (Mainz, Germany). The DAKO rabbit anti-human IgG antibody (Ab) conjugated to horseradish peroxidase (HRP) and goat anti-mouse Ig-HRP Ab were from Agilent Technologies Ireland, Ltd. (Cork, Ireland). Immoblion-P 0.45 µm polyvinylidene difluoride (PVDF) membrane was from Merck Millipore (Cork, Ireland). Purified LTA from *S. aureus* was purchased from InvivoGen (Toulouse, France). Pure, unlabelled lectins were purchased from EY Labs (San Mateo, CA, USA) or Vector Laboratories Inc. (Burlingame, CA, USA) Neoglycoconjugates were purchased from Dextra Laboratories Ltd. (Reading, UK) and IsoSep AB (Tullinge, Sweden) or synthesised in house [65] (Tables S3 and S4). Anti-PNAG mAb (F598) was as previously generated in the Pier lab [66]. All other reagents were purchased from Sigma-Aldrich Co. unless otherwise stated and were of the highest grade available.

### 4.2. Bacterial Strains and Culture

The *S. aureus* and *A. baumannii* strains used in this study (Table 1) are detailed in Table S1. All bacteria were grown on BHI agar. Agar was supplemented with tetracycline (5 μg/mL) for all *S. aureus* ∆*ica* strains. Bacteria were grown overnight (17 h) in 5 mL cultures at 37 °C with shaking at 180 rpm in BHI, BHI supplemented with 1% (*w/v*) glucose (BHI glucose) or BHI supplemented with 4% (*w/v*) NaCl (BHI NaCl) where indicated.

### 4.3. Biofilm Assays

Overnight cultures grown in BHI media were adjusted with BHI media to an absorbance at 595 nm of 1.0 and diluted 1:200 with BHI, BHI glucose or BHI NaCl. After mixing, 100 µL was placed in each well of Nunclon (∆ surface) tissue culture-treated 96-well microtitre plates in triplicate per sample, incubated at 37 °C for 24 h, washed three times in a basin of deionised water and dried at 80 °C for up to 2 h. Crystal violet solution (0.4% (*w/v*) crystal violet in distilled water, 100 µL) was added to each well and incubated for 5 min at room temperature. The wells were then washed three times with sterile water and 100 µL of 5% (*v/v*) acetic acid was added to the wells. Absorbance was measured at 490 nm on a SpectraMax M5e microplate reader (Molecular Devices, Inc.). Experiments were carried out in triplicate and the average absorbance and standard error were calculated using Excel v.2010 (Microsoft). To assess biofilm formation by *A. baumannii*, bacteria were grown overnight in BHI media at 37 °C with shaking at 180 rpm. Overnight cultures were diluted 1:200 in BHI glucose in 10 mL borosilicate glass culture tubes in a total volume of 2 mL. Cultures were incubated at 37 °C for 5 h with shaking at 270 rpm, then removed from the tubes and the tubes were washed three times with PBS and dried at 80 °C for 3 h. Crystal violet solution (3 mL) was added to the tubes for 10 min, then washed three times in water, dried at 80 °C for 3 h and the stained tubes imaged using a digital camera. Digital images were stored as .tif files.

### 4.4. Fluorescent Labeling of Bacteria

Bacterial labelling was carried out in the dark essentially as previously described [20], with some minor alterations. After overnight culture, bacteria were pelleted by centrifugation (5000× *g*, 5 min), washed three times in in Tris-buffered saline supplemented with Ca^2+^ and Mg^2+^ ions (TBS; 20 mM Tris-HCl, 100 mM NaCl, 1 mM CaCl_2_, 1 mM MgCl_2_, pH 7.2) and resuspended in 5 mL TBS. Bacteria were diluted with TBS to an absorbance at 595 nm of approximately 1.0. To determine the optimum dye concentration, each bacterial strain was incubated in the dark with a range of 5 to 50 µM SYTO^®^ 82 at 37 °C for 1 h with 180 rpm rotation. Following incubation, the fluorescently labelled cells were washed three times in TBS by resuspending bacteria in 1 mL TBS, centrifuging to pellet at 5000× *g* for 5 min and removing the supernatant to remove excess dye. Bacterial cells were finally resuspended to an approximate absorbance at 595 nm of 2.0 in 0.5 mL of TBS supplemented with 0.025% Tween-20 (TBS-T), and fluorescence was measured (λ_ex_ 541 nm, λ_em_ 560 nm) in a black microtitre plate using a SpectraMax M5e microplate reader (Figure S6). The optimum dye concentration was determined based on maximum fluorescence intensity obtained for the strain (5 μM SYTO^®^ 82 for *S. aureus* Mn8m and ∆*ica* mutant and *A. baumannii* WT and ∆*pga* mutant, and 10 μM for *S. aureus* 8325-4 WT and ∆*ica* mutant). For *S. aureus* BH1CC WT and ∆*ica* mutant, optimum dye concentration of 15 μM was selected based on optimum signal to noise ratio on the lectin microarray (Figure S7).

### 4.5. Assay for PNAG on Bacterial Cell Surface

Bacteria were grown overnight on BHI agar, inoculated in 5 mL of BHI glucose (*S. aureus* Mn8m and *A. baumannii*) or BHI NaCl (*S. aureus* 8325-4 and BH1CC) and grown overnight at 37 °C. Bacterial cells were washed by centrifugation at 5000× *g* for 5 min, resuspending the pellet with sterile TBS to an absorbance at 595 nm of approximately 1.0 (approximately 8 × 10^8^ cells/mL) in endotoxin-free water. Cells were killed by heating to 95 °C for 40 min. Heat-killed bacteria were then streaked on BHI agar plates and incubated overnight at 37 °C to confirm cell death by lack of growth. PVDF membrane (0.45 µm) was pre-treated for 15 s in methanol, soaked in TBS for 5 min and allowed to partially dry. PNAG (2 μL of 1 mg/mL) purified from *S. aureus* Mn8m (see below) and heat-killed bacteria (2 μL) were pipetted on to the activated membrane in triplicate and allowed to dry. Membranes were then incubated for 1 h in 5% (*w/v*) skimmed milk in TBS at room temperature, solution was drawn off and human IgG1 anti-PNAG mAb (F598 [66]) (800 µg/mL diluted in TBS 0.0001% Tween^®^ 20, 1% skimmed milk) was added to the membrane and incubated for 1 h. The membrane was then washed three times for 5 min each in TBS 0.0001% Tween^®^ 20 and once in TBS for 5 min. HRP-conjugated rabbit anti-human IgG antibody (200 µg/mL TBS with 0.0001% Tween^®^ 20 and 1% skimmed milk) was applied to the membrane, incubated at room temperature for 1 h and the membrane washed as above. For development, a chemiluminescent substrate for the detection of HRP activity (Pierce™ ECL Western blotting substrate) was added to the membrane for 1 min before visualisation using a chemiluminescent camera (Alpha Innotech FluorChem FC2 Imaging System). Images were stored digitally as .tif files.

### 4.6. Purification and Characterization of PNAG from S. aureus Mn8m

PNAG was purified from 16 L of the PNAG-overproducer *S. aureus* Mn8m culture by ethanol precipitation; enzymatic digestions were followed by size exclusion chromatography as previously described [39]. 1D ^1^H NMR spectra were acquired, also as previously described [39]. For the detection of LTA and peptidoglycan in PNAG, a dot blot was carried out as described above except 2 μL of 1 mg/mL PNAG *S. aureus* Mn8m and 2 μL dilutions of purified LTA purified from *S. aureus* (SA-LTA) were spotted on to the activated PVDF membrane. For a positive peptidoglycan sample, 2 µL of heat-killed *S. aureus* Mn8m (8 × 10^8^ cells/mL) was spotted on the membrane as a positive control. The membrane was blocked as described above and incubated with anti-LTA mAb (1:50 dilution in TBS-T 0.001%, 1% skimmed-milk) or mouse IgG1 anti-peptidoglycan mAb (1:50 dilution in TBS with 0.0001% Tween^®^ 20 and 1% skimmed milk) for 1 h at room temperature. The membrane was then washed three times for 5 min each in TBS 0.0001% Tween^®^ 20 and once in TBS for 5 min followed by incubation with HRP-labelled goat anti-mouse IgG antibody (200 µg/mL in TBS-T 0.001%, 1% skimmed-milk) for 1 h at room temperature. The membrane was washed three times in TBS-T and finally in TBS for 5 min. HRP activity was detected as described above and the image was quantified by densitometry using ImageJ and comparing to the standard curve generated by known concentrations of SA-LTA.

### 4.7. Fluorescent Labeling of PNAG

The entire procedure was carried out in the dark. PNAG was initially solubilised at 4 mg/mL in 5 M HCl and pH was immediately adjusted to 7.0 with 5 M NaOH. The solubilised PNAG was then diluted to 2 mg/mL in 0.1 M sodium borate (final concentration), pH 8.0, and 0.1 mg of AF555 carboxylic acid succinimidyl ester fluorescent label in 10 μL DMSO was added. The mixture was incubated at 25 °C for 2 h in the dark and the AF555-labelled PNAG (PNAG-AF555) was then purified using a 3 kDa MWCO centrifugal filter with three exchanges of 300 μL phosphate-buffered saline (PBS), pH 7.4. PBS (100 μL) was then added to the filter retentate and recovered according to manufacturer’s instructions. PNAG-AF555 (approximately 5 mg/mL) was not directly quantified after labelling but was titrated for optimal incubation concentration on lectin microarrays as detailed below.

### 4.8. Lectin and Carbohydrate Microarray Construction

Lectin and carbohydrate microarrays were prepared essentially as previously described [20,65], with minor modifications. In brief, a panel of lectins of known specificities was printed at 0.5 mg/mL in PBS, pH 7.4, supplemented with 1 mM of their respective haptentic monosaccharides (Table S2) in replicates of six per probe per subarray and eight replicate subarrays per microarray slide. For carbohydrate microarrays, neoglycoconjugates and glycoproteins were printed at 1 mg/mL in PBS across two paired microarrays, A and B, with 15 of the same probes in the same print position to facilitate later data normalisation across the paired microarrays (Tables S3 and S4). Probes (lectins, NGCs and glycoproteins) were printed on Nexterion^®^ slide H microarray slides using a SciFlexArrayer S3 (Scienion, Berlin, Germany) under constant 60% (+/−2%) humidity at 20 °C. For each slide, features of approximately 1 nL were printed in replicates of six per probe per subarray and eight replicate subarrays per microarray slide. Following printing, slides were placed in a humidity chamber overnight at room temperature. Slides were then blocked with 100 mM ethanolamine in 50 mM sodium borate, pH 8.0, for 1 h at room temperature. Slides were washed three times with PBS with 0.05% Tween^®^ 20 (PBS-T), and once with PBS. Slides were dried by centrifugation (1500 rpm, 5 min) and stored at 4 °C sealed with desiccant until use. Validation of lectin printing and retained function on the microarray surface was carried out by incubating one microarray from each batch with a panel of AF555-labelled glycoproteins (fetuin, asialofetuin, invertase, RNase B and alpha-1-acid glycoprotein) and the neoglycoconjugate GlcNAc-BSA (each incubated at 1 µg/mL in TBS-T). Validation of neoglycoconjugate and glycoprotein printing and accessibility of the presented carbohydrates on the microarray surface was done by incubating one microarray from each batch with a panel of TRITC-labelled lectins (WGA, MAA, AIA, Con A, PHA-E, GS-II and SBA, each incubated at 5 µg/mL).

### 4.9. Microarray Incubation and Scanning

All microarray incubations were carried out in the dark. Labelled bacteria, PNAG, lectins or glycoproteins diluted in TBS-T were incubated on lectin or carbohydrate microarrays essentially as previously described [20] at 70 uL per well and incubated with gentle rotation (4 rpm) at 37 °C for 1 h. Incubation chambers were disassembled under TBS-T, washed twice in TBS-T for 2 min each wash in a Coplin jar and once with TBS. Microarray slides were dried by centrifugation (1500 rpm, 5 min) and imaged immediately after incubation and washing by scanning in an Agilent G2505B microarray scanner equipped with a 543 nm laser (90% PMT, 5 μm resolution). Images were stored digitally as .tif files (Figure S13). Experiments were carried out in technical triplicate with sample incubation on one microarray slide considered as one experimental replicate. All bacterial strains were initially titrated on each microarray platform by incubating dilutions of stained bacteria at absorbance at 595 nm of 2.0 with TBS-T in a final 70 uL per well volume. As *S. aureus* BH1CC had the lowest fluorescence following staining compared to the other *S. aureus* strains and *A. baumannii*, the optimal dilution of 50 μL to a final volume of 70 μL with TBS-T of *S. aureus* BH1CC WT (maximal signal intensity with low background) was selected for use as the dilution for all bacterial strains for consistency (1.1 × 10^9^ cells/mL). For PNAG-AF555, the optimal dilution of 0.2 µL stock per mL of TBS-T was used. For inhibition assays, varying concentrations of the sugar were co-incubated with PNAG-AF555 in different subarrays and compared to uninhibited binding on the same microarray slide.

### 4.10. Microarray Data Extraction and Analysis

Data extraction was performed essentially as previously described [20,50]. Local-background-subtracted median feature intensity data (F543median-B543) was analysed and the median of six replicate spots per subarray was handled as a single data point for graphical and statistical analysis. For lectin microarray analysis, data were normalized to the per-subarray mean total intensity value of three replicate microarray slides, and binding data was presented as a bar chart of the average intensity of three experimental replicates with error bars of +/−1 SD of the mean. For carbohydrate microarray analysis, the same process was carried out as for lectin microarray analysis except that total per-subarray intensity was normalised to the common 15 probes across the paired A and B microarrays [50]. IC_50_ values were generated using GraphPad Prism v.8.3.1 (GraphPad Software, San Diego, CA, USA) using a nonlinear fit of percentage inhibition versus Log_10_ inhibitor concentration. Unsupervised hierarchical clustering of binding data was carried out using Hierarchial Clustering Explorer v3.0 (http://www.cs.umd.edu/hcil/hce/hce3.html; National Institutes of Health, Bethesda, MD, USA). Normalised microarray data was scaled to the maximum signal intensity per sample and the binding patterns were clustered using no pre-filtering, complete linkage and Euclidean distance. T-tests comparing mutant and WT binding were carried out using normalised data, 2-tailed and unequal variance.

## 5. Conclusions

In summary, this work showed that sWGA and WGA selectively bind to PNAG in a carbohydrate-dependent manner, that the main accessible surface component of MSSA strain Mn8m was PNAG while PNAG only partially covered the surface of MSSA strain 8325-4, and that PNAG was not the main accessible surface molecule on *A. baumannii*. This study is also the first to report specific carbohydrate ligands for whole *S. aureus* and *A. baumannii* bacteria, and a GlcNAc-binding lectin function was shown to have a role in PNAG surface presentation or biofilm assembly for *A. baumannii*. Together, these data indicated that PNAG surface presentation and accessibility differed between these Gram-negative and Gram-positive species, and that PNAG affected the presentation and accessibility of other surface molecules on *A. baumannii* cells. Based on these data, we suggest that PNAG may fulfil different biological and/or physical roles depending on the surface presentation. These findings will help to advance our understanding of host–pathogen interactions, biofilm assembly and mechanisms of pathogenesis.

## Figures and Tables

**Table 1:** Major biofilm types and effects of glucose and NaCl on biofilm formation by selected bacterial strains. All reports for *S. aureus* are based on biofilm assays carried out on hydrophilic 96-well plates [9,25,26,27,29,30,31]. Reports for *A. baumannii* are based on biofilm formation on borosilicate glass tubes [28]. n.d.—not determined in reports to date. n.a.—not applicable.

Species and Strain	Sensitivity	Additive	Biofilm Effect	Major Biofilm Type		
				Protein	eDNA	PNAG
*S. aureus* 8325-4	MSSA	Glc	↑			✓
		NaCl	↑			✓
*S. aureus* Mn8m	MSSA	Glc	↑			✓
		NaCl	n.d.	n.d.	n.d.	n.d.
*S. aureus* BH1CC	MRSA	Glc	↑	✓	✓	
		NaCl	↓	n.d.	n.d.	n.d.
*A. baumannii* S1	n.d.	Glc	n.d.			✓
		NaCl	n.d.	n.d.	n.d.	n.d.

## Supplementary Materials

Supplementary materials can be found at https://www.mdpi.com/1422-0067/21/7/2465/s1.

## Abbreviations

PNAG	poly-*N*-acetylglucosamine
MSSA	methicillin-sensitive *S. aureus*
MRSA	methicillin-resistant *S. aureus*
WGA	Wheatgerm agglutinin
sWGA	Succinylated WGA
eDNA	extracellular DNA
mAb	Monoclonal antibody
WT	Wild type
GlcNAc	*N*-acetylglucosamine
LTA	Lipoteichoic acid
Man	Mannose
IC_50_	half maximal inhibitory concentration
HPA	*Helix pomatia* agglutinin
GSL-II	*Griffonia simplicifolia* lectin-II
DSA	*Datura stramonium* agglutinin
AAL	*Aleuria aurantia* lectin
MPA	*Maclura pomifera* agglutinin
SSEA3	stage specific embryonic antigen 3
DBA	*Dolichos biflorus* agglutinin
ACA	*Amaranthus caudatus* agglutinin
AMA	*Arum maculatum* agglutinin
LacNAc	*N*-acetyllactosamine
CPA	*Cicer arietinum* agglutinin
VRA	*Vigna radiata* agglutinin
LPS	lipopolysaccharide
CPS	Capsular polysaccharide
3SLac	3′sialyllactose
6SLac	6′sialyllactose
LNT	lacto-*N*-tetraose
FnBP	fibronectin-binding protein
Lex	Lewis x
BGA	Blood group A
BGB	Blood group B
BHI	Brain Heart Infusion
HRP	Horseradish peroxidase
