# Supplementary material for: Glycomics Microarrays Reveal Differential In Situ Presentation of the Biofilm Polysaccharide Poly-N-acetylglucosamine on Acinetobacter baumannii and Staphylococcus aureus Cell Surfaces
Source: Int J Mol Sci. 2020 Apr 2;21(7):2465. doi: 10.3390/ijms21072465 (PMC7177611; doi:10.3390/ijms21072465)
Supplement: Supplementary file 1 [file ijms-21-02465-s001.pdf]

Results and Discussion

Bacterial Strain Selection and Verification of Biofilm Production.

The MSSA strains 8325-4 and Mn8m were selected as Gram-positive organisms that produce PNAG-predominant biofilm (Table 1 and Table S1) [1,2]. In *S. aureus*, PNAG is produced by proteins encoded in the *ica* operon and thus the Δ*ica* mutants of the *S. aureus* strains [3,4] were included in this study. The MRSA clinical isolate strain BH1CC has an *ica* operon but does not produce PNAG. Instead eDNA is the main biofilm component [5]. *S. aureus* BH1CC wild type (WT) and the Δ*ica* mutant were included for comparison with the MSSA strains. In some species of Gram-negative bacteria, PNAG is synthesised by proteins produced by the *pga* operon, so the PNAG-producing clinical isolate *A. baumannii* strain S1 WT and its Δ*pga* mutant [6] were also included. Anti-PNAG monoclonal antibody (mAb) was used in a dot blot assay to confirm that the PNAG-producing strains *S. aureus* 8325-4, *S. aureus* Mn8m and *A. baumannii* S1 cultured under biofilm-promoting conditions retained PNAG *in situ* on the cell surface under experimental conditions, while the Δ*ica* and Δ*pga* mutants did not present any PNAG as expected (Figure 1b).

It is critical to initially verify that the selected strains and corresponding PNAG-deficient Δ*ica* or Δ*pga* mutants behaved as previously reported, so crystal violet biofilm assays were performed on these strains (Table 1 and Figure S1). All bacterial strains were grown in the presence of 1% glucose supplemented into the growth media (Figure S1) as glucose promotes PNAG-mediated biofilm formation in the MSSA strains but in MRSA clinical isolates, glucose promotes biofilm formation *via* an *ica*-independent mechanism that involves extracellular surface proteins, such as FnBPAB, and eDNA [4,7,8]. Four percent NaCl was also added to the growth media of MRSA BH1CC as it promotes *icaA* transcription but does not promote biofilm formation [7]. As a comparison, the MSSA strain 8325-4 was also grown in the presence of NaCl which increases PNAG-mediated biofilm formation in this strain [9].

Addition of glucose to brain heart infusion (BHI) media increased biofilm formation by *S. aureus* 8325-4 WT by approximately 165% in comparison to *S. aureus* 8325-4 grown in BHI media (Figure S1A) and by 311% with the addition of NaCl to BHI. *S. aureus* 8325-4 Δ*ica* decreased biofilm formation by approximately 73%, 66% and 96% compared to the WT grown in the same media, respectively, which confirmed PNAG-mediated biofilm formation (Figure S1A). *S. aureus* Mn8m WT biofilm was increased slightly (approximately 5%) by the addition of glucose compared to BHI alone, while *S. aureus* Mn8 Δ*ica* biofilm formation decreased by approximately 92% in BHI and 86% in BHI glucose compared to the WT strain under the same conditions (Figure S1B), which indicated that PNAG was the major contributor to biofilm formation of this MSSA strain. *S. aureus* BH1CC had increased biofilm formation (approximately 62%) when cultured in BHI glucose compared to BHI alone but the addition of NaCl decreased biofilm formation by 90% (Figure S1C). *S. aureus* BH1CC Δ*ica* decreased biofilm formation slightly, by approximately 16%, 13% and 2% of the WT biofilm formed when grown in BHI alone, BHI glucose and BHI NaCl, respectively, but these differences were not significant. This indicated that *S. aureus* BH1CC biofilm formation was not PNAG-dependent. As *A. baumannii* preferentially forms biofilm on glass [6], *A. baumannii* S1 WT and Δ*pga* were grown in the presence of BHI supplemented with 1% Glc in a borosilicate glass culture tube with vigorous shaking (Figure S1D). Crystal violet staining was more intense on the *A. baumannii* S1 WT glass culture tube compared to the Δ*pga* mutant which indicated that while the majority component of *A. baumannii* biofilm was PNAG, other macromolecules (e.g. protein or eDNA) are also components of its biofilm.

Overall, these data confirmed that *S. aureus* strains 8325-4 and Mn8m WT had increased biofilm formation in the presence of glucose and/or NaCl and that this biofilm was primarily composed of PNAG, *S. aureus* BH1CC WT had increased biofilm formation in the presence of glucose and decreased or abolished biofilm in NaCl but PNAG was not involved in biofilm formation as expected and that PNAG contributed to *A. baumannii* S1 biofilm formation.

Surface PNAG Retention under Experimental Conditions.

For glycomic microarray analysis it is necessary to fluorescently label bacteria internally by optimising the dye concentrations for each strain under biofilm-promoting conditions (Figures S5–S8). To minimise signal quenching and potential interference of the free highly charged fluorescent molecules in bacterial interactions, the bacteria must be thoroughly washed after staining to remove excess dye [10]. Accordingly several wash conditions after SYTO®82 staining were assessed to determine the retention of PNAG on the bacterial surface (Figure S5). The positive reference of maximal PNAG retention (100%) was for *S. aureus* 8325-4 WT not washed after staining with SYTO® 82 with release of bound PNAG and detection of the released PNAG by anti-PNAG mAb. Washing three times and resuspension of the labelled cells in Tris buffered saline with Ca^{2+} and Mg^{2+} ions (TBS) supplemented with no or varying concentrations of detergent were compared (Figure S5). Although wash buffer with no detergent retained more cell-surface PNAG compared to wash buffer including detergent (approximately 60% retention), not including detergent in microarray incubations resulted in bacterial clumping. Hence washing the stained bacteria three times and resuspension in TBS supplemented with 0.025% Tween-20 (TBS-T) was selected for all microarray experiments.

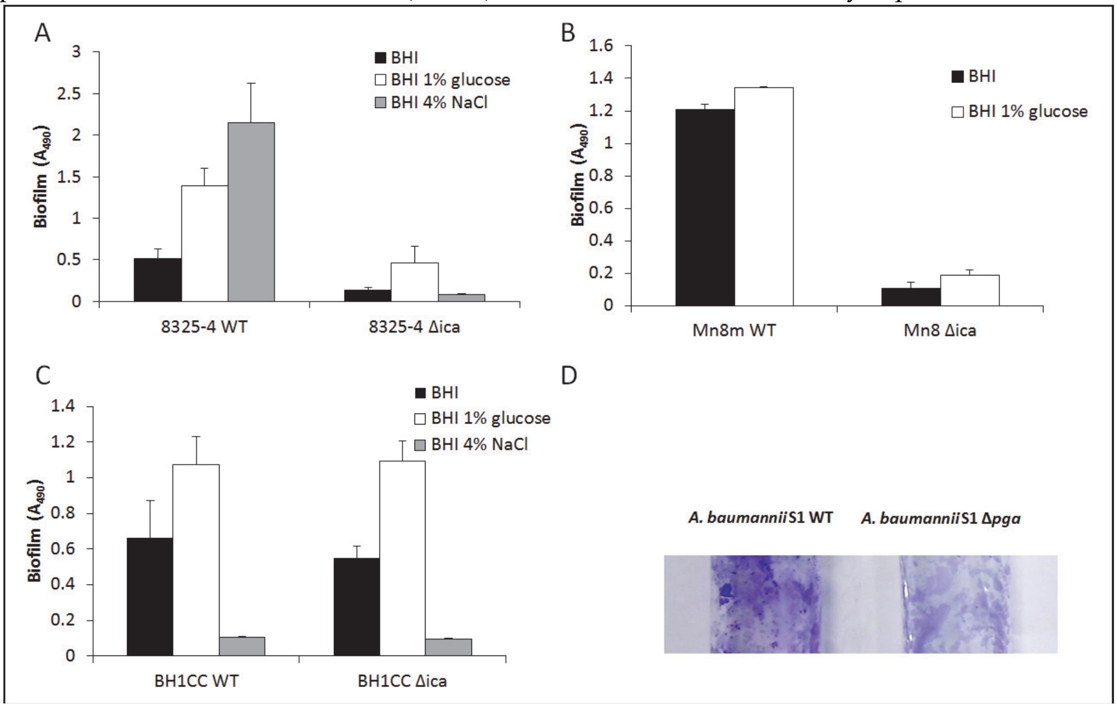

Figure S1. Biofilm assays for (**A**) *S. aureus* 8325-4 wild type (WT) and Δ*ica*, (**B**) *S. aureus* Mn8m WT and Δ*ica*, (**C**) *S. aureus* BH1CC WT and Δ*ica*, and (**D**) *A. baumannii* S1 WT and Δ*pga.* For (**A**), (**B**) and (**C**), bacteria were grown BHI, BHI supplemented with 1% glucose or 4% NaCl in a hydrophilic 96-well tissue culture-treated plate for 18 h. Biofilm was quantified by adding crystal violet and measuring the absorbance at 490 nm. Experiments were carried out in technical triplicates and data is presented as the mean of the three technical replicates of three experiments with error bars of +/-1 standard deviation (SD) of the mean. (**D**) Bacteria were grown for 18 h in borosilicate glass tubes. Tubes were washed and stained with crystal violet, washed with water, dried and imaged using a camera.

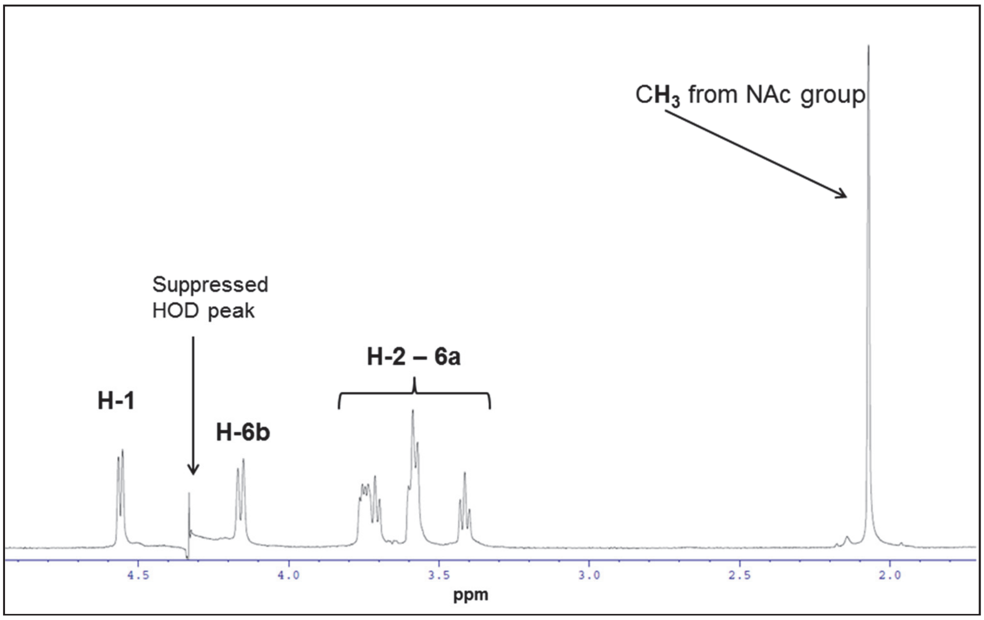

Figure S2. 600 MHz ^{1}H NMR spectra of *S. aureus* Mn8m PNAG in D_2O. Assignments as previously published [11].

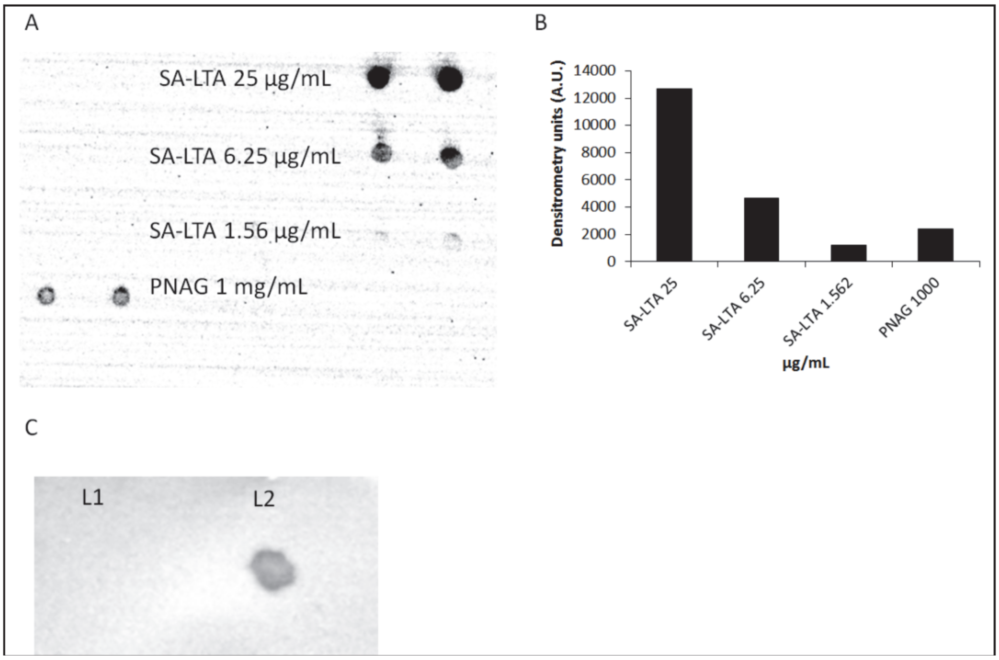

Figure S3. Dot blot assay for the detection of lipoteichoic acid (LTA) and peptidoglycan. (**A**) Dot blot assay of a standard curve of *S. aureus* LTA (SA-LTA) at 25, 6.25 and 1.56 µg/mL and partially purified PNAG (1 mg/mL). Black colour intensity represents anti-LTA antibody (Ab) binding. Spotting was carried out in duplicates. (**B**) Densitometry analysis of image (A) using ImageJ software. (**C**) Dot blot assay for peptidoglycan detection using an anti-peptidoglycan monoclonal Ab. L1 represents the PNAG preparation and L2 represents 8 x 10^8 cells/mL of heat killed *S. aureus*.

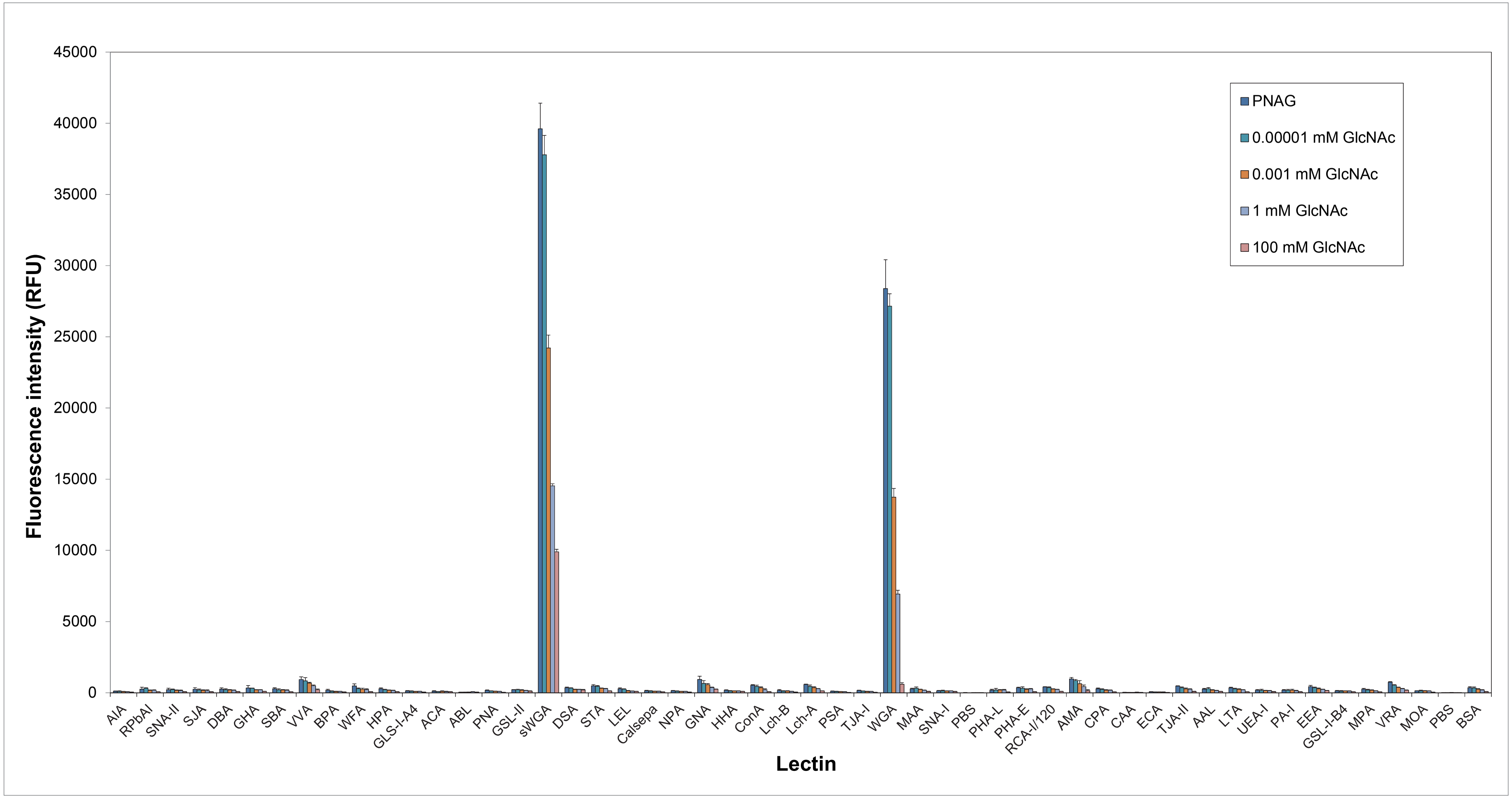

Figure S4. Fluorescently labelled PNAG incubated on the lectin microarray with co-incubation of different concentrations of GlcNAc as indicated in legend. Bars represent the mean of three experiments with error bars of +/- 1 SD of the mean.

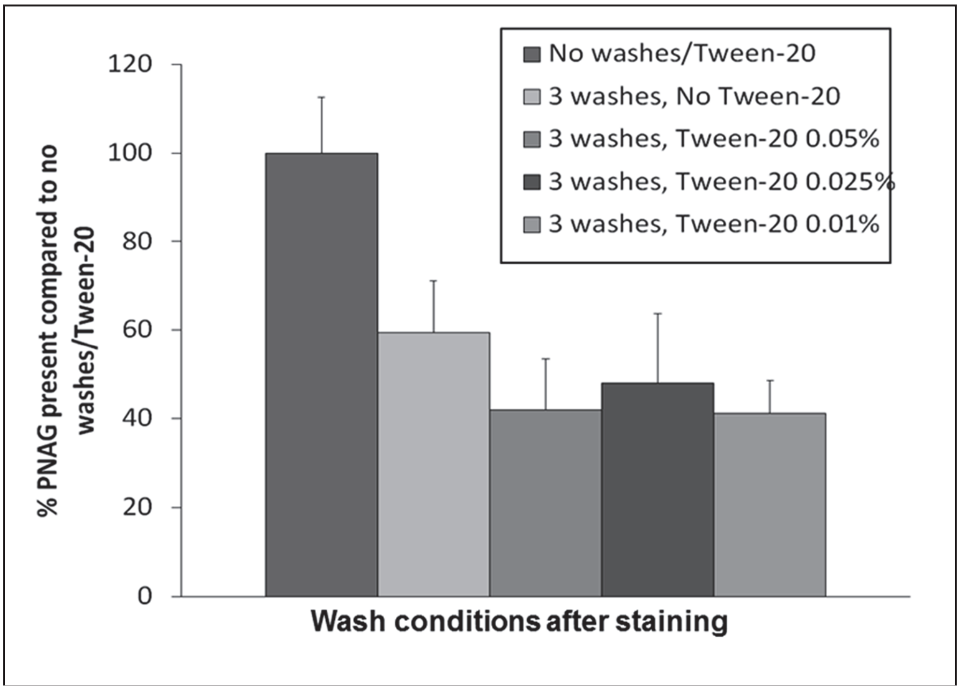

Figure S5. Detection of PNAG released from *S. aureus* 8325-4 WT surface with no washes and after three washes with no and varying Tween-20 concentrations. Anti-PNAG mAb binding was quantified by densitometry and the presence of PNAG was plotted as a relative percentage of no washing after staining (100%).

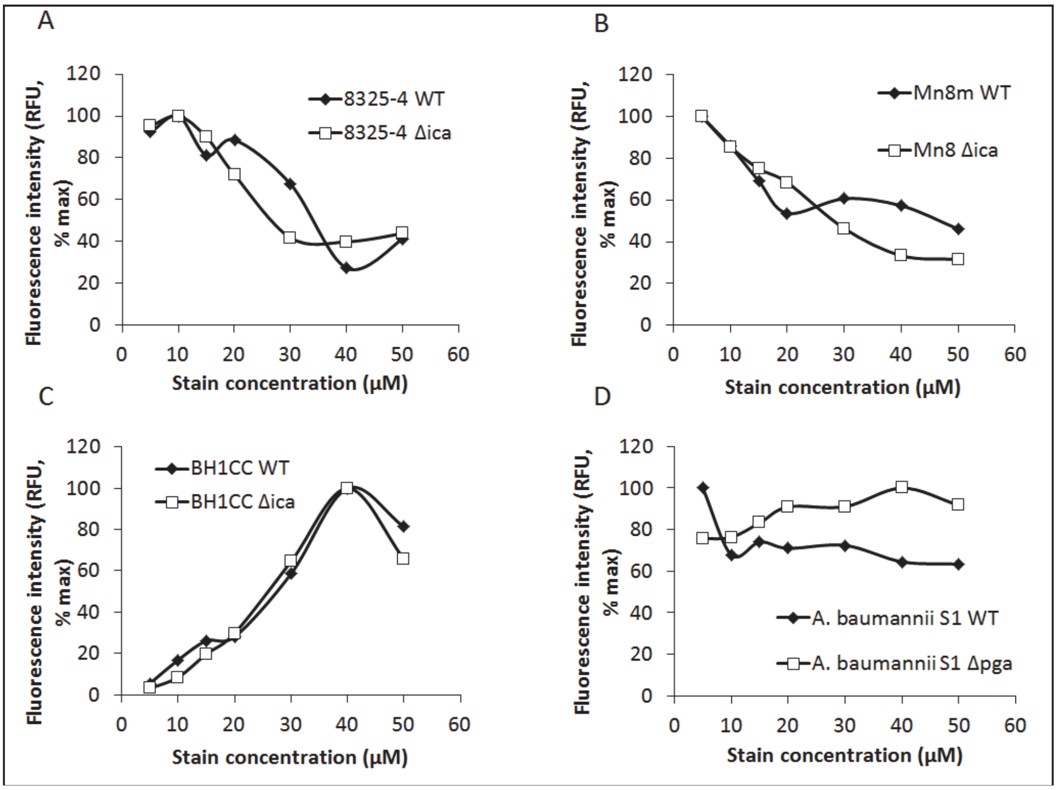

Figure S6. SYTO®82 concentration titration for bacterial fluorescence of (**A**) *S. aureus* 8325-4 WT and Δ*ica*, (**B**) *S. aureus* Mn8m WT and Δ*ica*, (**C**) *S. aureus* BH1CC WT and Δ*ica* and (**D**) *A. baumannii* S1 WT and Δ*pga*. For (**B**), (**C**) and (**D**), bacteria were grown overnight in BHI glucose, while (**A**) *S. aureus* 8325-4 was grown in BHI NaCl, and all strains were incubated with 5-50 µM SYTO®82. Fluorescence of the stained bacteria was measured at λ_{ex} 541 nm and λ_{em} 560 nm and plotted as a percentage of maximum fluorescence obtained for each strain.

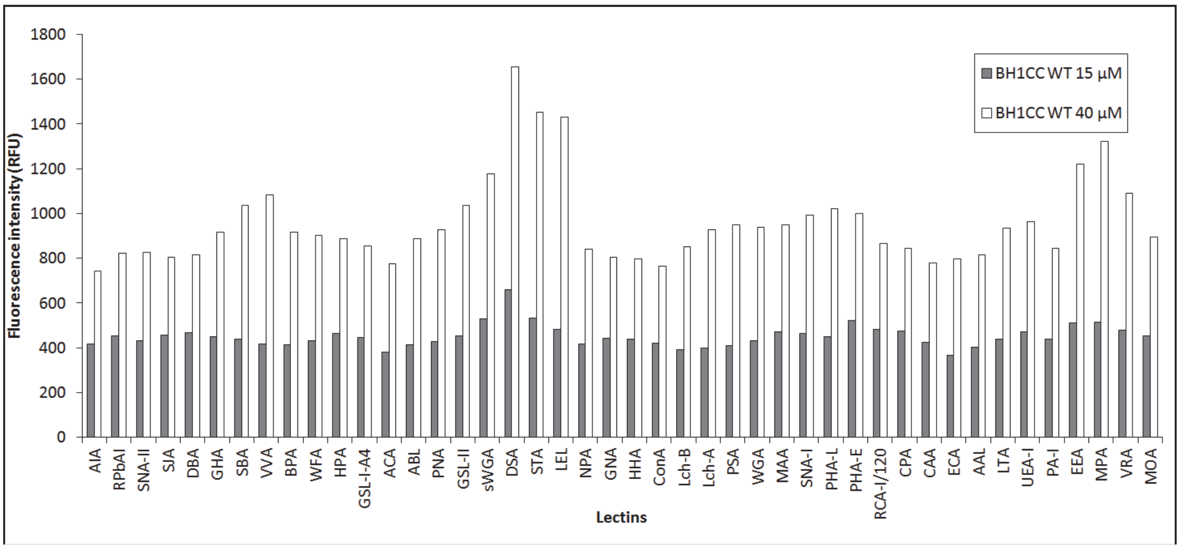

Figure S7. Lectin microarray background fluorescence of *S. aureus* BH1CC WT stained with 15 and 40 µM SYTO® 82. *S. aureus* BH1CC WT stained with 15 and 40 µM SYTO® 82 incubated on the lectin microarray and the local average background around each lectin represented as a bar chart. As 15 µM SYTO® 82 resulted in similar signal intensity with lower background compared to 40 µM, 15 µM was selected as the optimal concentration for staining *S. aureus* BH1CC WT and Δ*ica*.

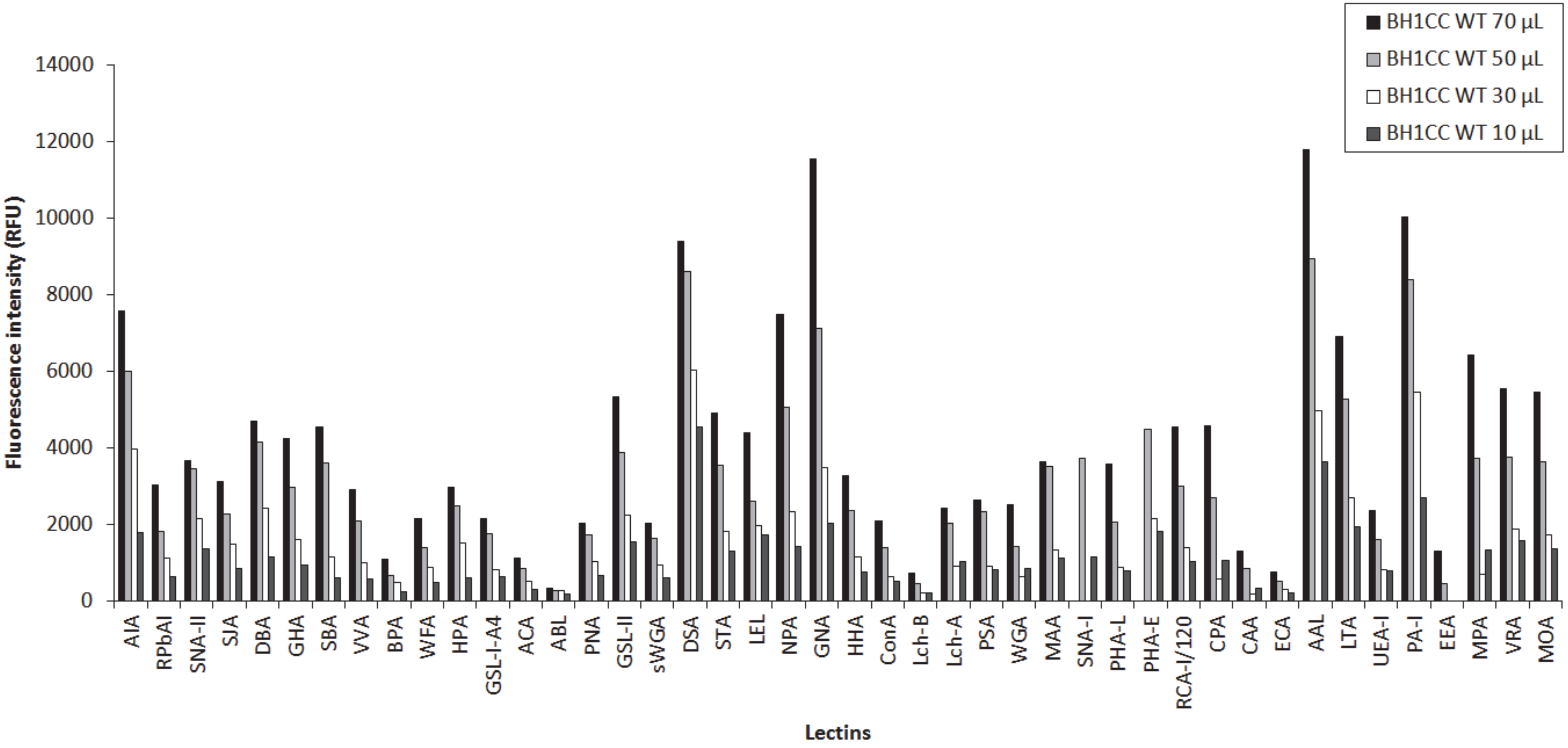

Figure S8. *S. aureus* BH1CC WT titration on the lectin microarray. *S. aureus* BH1CC was either not diluted (BH1CC WT 70 µL), 50 µL of the stained bacteria were diluted with TBS-T to a final volume of 70 µL (BH1CC WT 50 µL), 30 µL diluted to a final volume of 70 µL (BH1CC WT 30 µL) or 10 µL diluted to a final volume of 70 µL (BH1CC WT 10 µL) and incubated on the lectin microarray. Bars represent the binding intensity from one experiment and the median data from six technical replicates.

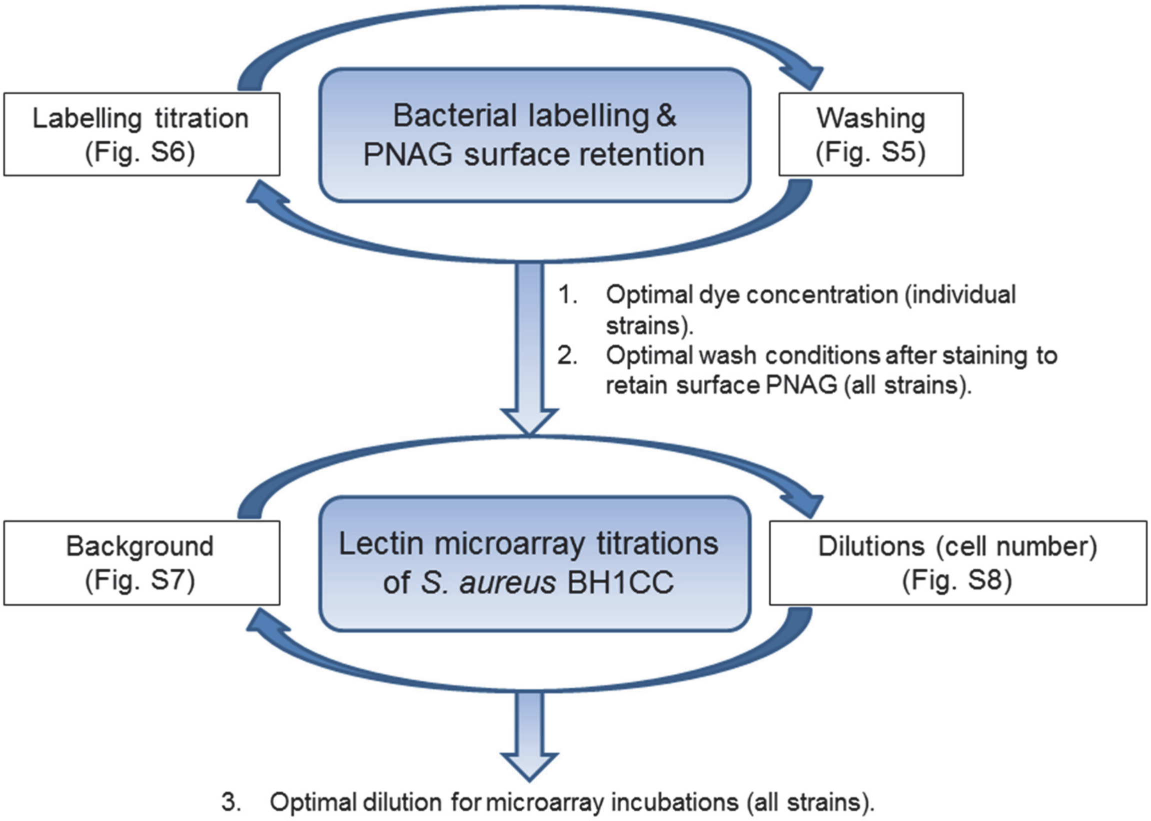

Figure S9. Flow chart summarising optimisation steps and outcomes from Figures S5 to S8.

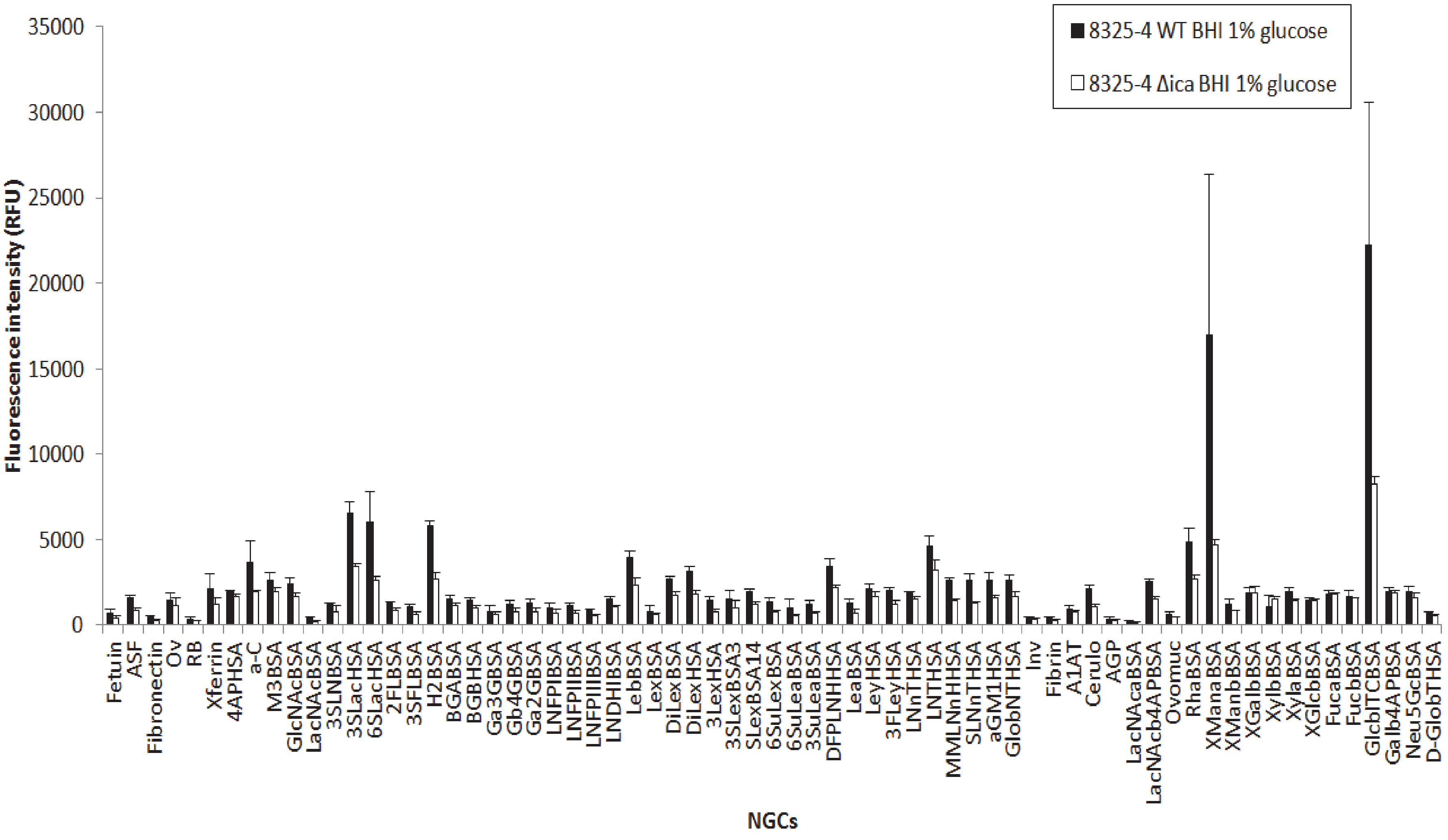

Figure S10. Carbohydrate microarray binding intensity profiles of *S. aureus* 8325-4 WT and Δ*ica* grown in BHI glucose. Bars represent the mean of three experiments with error bars of +/- 1 SD of the mean.

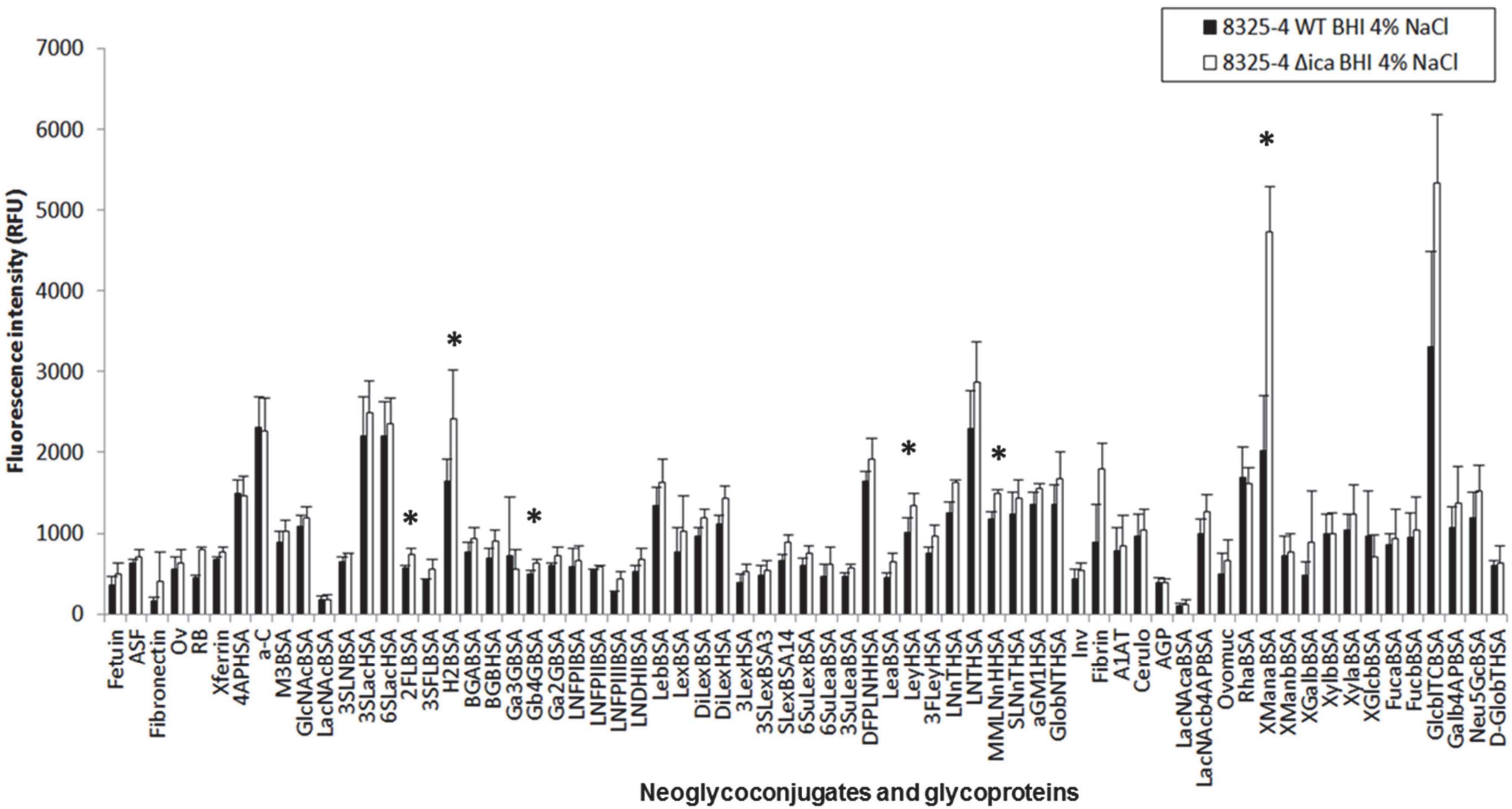

Figure S11. Carbohydrate microarray binding intensity profiles of *S. aureus* 8325-4 WT and Δ*ica* grown in BHI NaCl. Bars represent the mean of three experiments with error bars of +/- 1 SD of the mean. * represents significant difference ($p \leq 0.05$, calculated by student's t test, two tailed) in binding between WT and Δ*ica*.

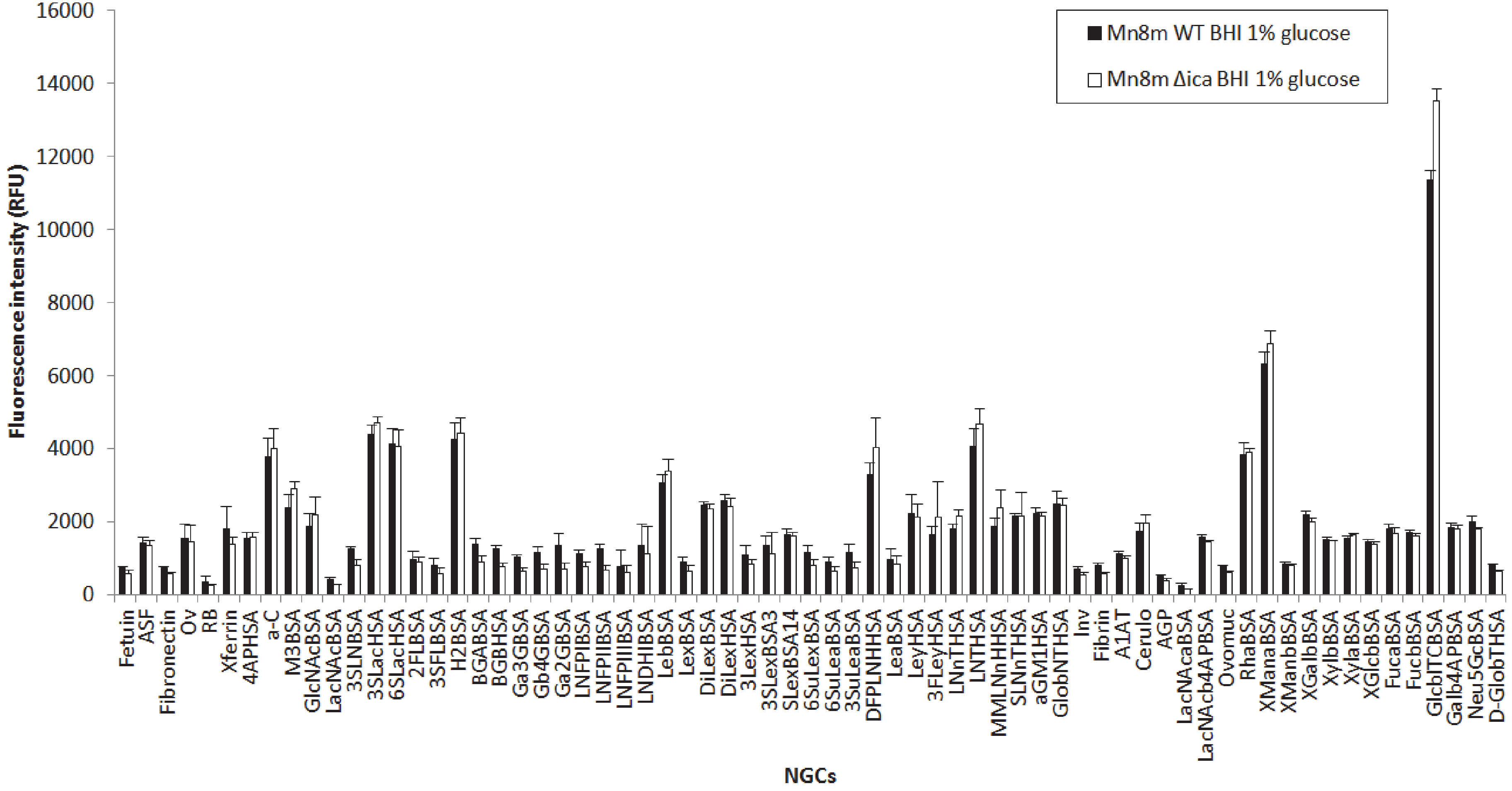

Figure S12. Carbohydrate microarray binding intensity profiles of *S. aureus* Mn8m WT and Δ*ica* grown in BHI glucose. Bars represent the mean of three experiments with error bars of +/- 1 SD of the mean.

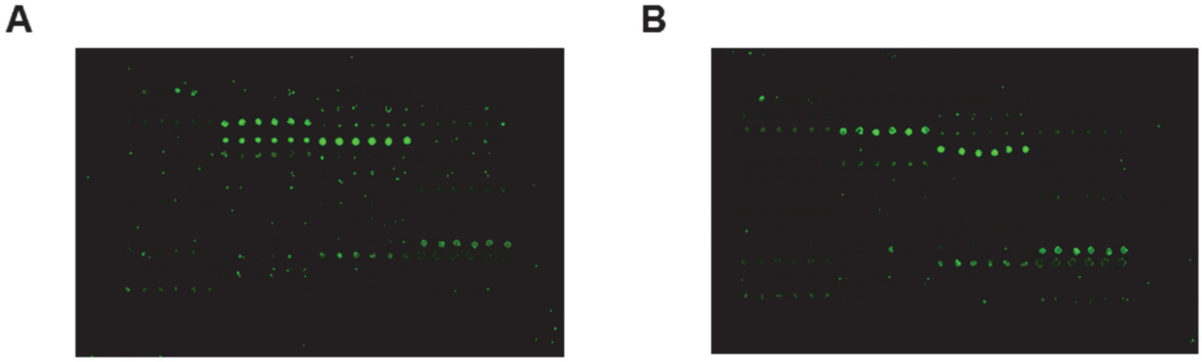

Figure S13. Example of lectin microarray subarrays incubated with fluorescently stained bacteria. (**A**) *S. aureus* Mn8m WT. (**B**) *S. aureus* Mn8m Δica mutant.

Table S1. The origins of bacterial strains used in this study.

Bacteria Strains	Details	Reference
S. aureus 8325-4 WT	8325 derivative cured of prophages. 11-bp deletion in rsbU	[1]
S. aureus 8325-4 Δ*ica*	*icaADBC*::Tr^r isogenic mutant of 8325-4	[4]
S. aureus Mn8m WT	Chemostat derived mutant of Mn8 (toxic shock syndrome isolate). Biofilm positive.	[2]
S. aureus Mn8 Δ*ica*	*icaADBC*::Tr^r isogenic mutant of Mn8	[3]
S. aureus BH1CC WT	MRSA clinical isolate. Biofilm positive. SCCmec type, MLST type 8, clonal complex 8. Isolate from Beaumont Hospital, Dublin, Ireland.	[7]
S. aureus BH1CC Δ*ica*	*icaADBC*::Tr^r isogenic mutant of BH1CC	[4]
A. baumannii S1 WT	Clinical isolate. Mucoid phenotype. Biofilm positive.	[6]
A. baumannii S1 Δ*pga*	S1 derivative with in-frame deletion of *pgaABC*	[6]

Table S2. Lectins printed, their binding specificities, their simple print sugars (1 mM) and the supplying company. Binding specificity is reported recognition based on literature consensus or experimental evidence generated within our laboratory.

Abbreviation	Source	Species	Common Name	General Binding Specificity	Print Sugar	Supplier
AIA, Jacalin	Plant	*Artocarpus integrifolia*	Jack fruit lectin	Gal, Gal-β-(1,3)-GalNAc (sialylation independent)	Gal	EY Labs
RPbAI	Plant	*Robinia pseudoacacia*	Black locust lectin	Gal	Gal	EY Labs
SNA-II	Plant	*Sambucus nigra*	Sambucus lectin-II	Gal/GalNAc	Gal	EY Labs
SJA	Plant	*Sophora japonica*	Pagoda tree lectin	β-linked GalNAc	Gal	EY Labs
DBA	Plant	*Dolichos biflorus*	Horse gram lectin	GalNAc	Gal	EY Labs
GHA	Plant	*Glechoma hederacea*	Ground ivy lectin	GalNAc	Gal	EY Labs
SBA	Plant	*Glycine max*	Soy bean lectin	GalNAc	Gal	EY Labs
VVA	Plant	*Vicia villosa*	Hairy vetch lectin	GalNAc	Gal	EY Labs
BPA	Plant	*Bauhinia purpurea*	Camels foot tree lectin	GalNAc/Gal	Gal	EY Labs
WFA	Plant	*Wisteria floribunda*	Japanese wisteria lectin	GalNAc/sulfated GalNAc	Gal	EY Labs
HPA	Animal	*Helix pomatia*	Edible snail lectin	α-linked GalNAc	Gal	EY Labs
GSL-I-A4	Plant	*Griffonia simplicifolia*	Griffonia isolectin I A4	GalNac	Gal	EY Labs
ACA	Plant	*Amaranthus caudatus*	Amaranthin	Sialylated/Gal-β-(1,3)-GalNAc	Lac	Vector Labs
ABL	Fungus	*Agaricus bisporus*	Edible mushroom lectin	Gal-β(1,3)-GalNAc, GlcNAc	Lac	EY Labs
PNA	Plant	*Arachis hypogaea*	Peanut lectin	Gal-β-(1,3)-GalNAc	Lac	EY Labs
GSL-II	Plant	*Griffonia simplicifolia*	Griffonia lectin-II	GlcNAc	GlcNAc	EY Labs
sWGA	Plant	*Triticum vulgaris*	Succinyl WGA	GlcNAc	GlcNAc	EY Labs
DSA	Plant	*Datura stramonium*	Jimson weed lectin	GlcNAc	GlcNAc	EY Labs
STA	Plant	*Solanum tuberosum*	Potato lectin	GlcNAc oligomers	GlcNAc	EY Labs
LEL	Plant	*Lycopersicum eculentum*	Tomato lectin	GlcNAc-β-(1,4)-GlcNAc	GlcNAc	EY Labs
NPA	Plant	*Narcissus pseudonarcissus*	Daffodil lectin	α-(1,6)-Man	Man	EY Labs
GNA	Plant	*Galanthus nivalis*	Snowdrop lectin	Man-α-(1,3)-	Man	EY Labs
HHA	Plant	*Hippeastrum hybrid*	Amaryllis agglutinin	Man-α-(1,3)-Man-α-(1,6)-	Man	EY Labs
ConA	Plant	*Canavalia ensiformis*	Jack bean lectin	Man, Glc, GlcNAc	Man	EY Labs

Lch-B	Plant	*Lens culinaris*	Lentil isolectin B	Man, core fucosylated, agalactosylated biantennary *N*-glycans	Man	EY Labs
Lch-A	Plant	*Lens culinaris*	Lentil isolectin A	Man/Glc	Man	EY Labs
PSA	Plant	*Pisum sativum*	Pea lectin	Man, core fucosylated trimannosyl *N*-glycans	Man	EY Labs
TJA-I	Plant	*Trichosanthes japonica*	Trichosanthes japonica agglutinin I	NeuAc-α-(2,6)-Gal-β-(1,4)-GlcNAc	Lac	Medicago
WGA	Plant	*Triticum vulgaris*	Wheat germ agglutinin	NeuAc/GlcNAc	GlcNAc	EY Labs
MAA	Plant	*Maackia amurensis*	Maackia agglutinin	Sialic acid-α-(2,3)-linked	Lac	EY Labs
SNA-I	Plant	*Sambucus nigra*	Sambucus lectin-I	Sialic acid-α-(2,6)-linked	Lac	EY Labs
PHA-L	Plant	*Phaseolus vulgaris*	Kidney bean leukoagglutinin	Tri- and tetraantennary β-Gal/Gal-β-(1,4)-GlcNAc	Lac	EY Labs
PHA-E	Plant	*Phaseolus vulgaris*	Kidney bean erythroagglutinin	Biantennary with bisecting GlcNAc,β-Gal/Gal-β-(1,4)-GlcNAc	Lac	EY Labs
RCA-I/120	Plant	*Ricinus communis*	Castor bean lectin I	Gal-β-(1,4)-GlcNAc	Gal	Vector Labs
AMA	Plant	*Arum maculatum*	Lords and ladies lectin	Gal-β-(1,4)-GlcNAc	Lac	EY Labs
CPA	Plant	*Cicer arietinum*	Chickpea lectin	Complex oligosaccharides	Lac	EY Labs
CAA	Plant	*Caragana arborescens*	Pea tree lectin	Gal-β-(1,4)-GlcNAc	Lac	EY Labs
ECA	Plant	*Erythrina cristagalli*	Cocks comb/coral tree lectin	Gal-β-(1,4)-GlcNAc oligomers	Lac	EY Labs
TJA-II	Plant	*Trichosanthes japonica*	Trichosanthes japonica agglutinin II	Fuc-α-(1,2)-Gal-β-(1,4)-GlcNAc	Lac	Medicago
AAL	Fungi	*Aleuria aurantia*	Orange peel fungus lectin	Fuc-α-(1,6)-linked, Fuc-α-(1,3)-linked	Fuc	Vector Labs
LTA	Plant	*Lotus tetragonolobus*	Lotus lectin	Fuc-α-(1,3)-linked	Fuc	EY Labs
UEA-I	Plant	*Ulex europaeus*	Gorse lectin-I	Fuc-α-(1,2)-Gal	Fuc	EY Labs
PA-I	Bacteria	*Pseudomonas aeruginosa*	Pseudomonas lectin	Terminal α-linked Gal, Gal derivatives	Gal	EY Labs
EEA	Plant	*Euonymous europaeus*	Spindle tree lectin	Terminal α-linked Gal	Gal	EY Labs
MPA	Plant	*Maclura pomifera*	Osage orange lectin	Terminal α-linked Gal	Gal	EY Labs
VRA	Plant	*Vigna radiata*	Mung bean lectin	Terminal α-linked Gal	Gal	EY Labs
MOA	Fungus	*Marasmius oreades*	Fairy ring mushroom lectin	Terminal α-linked Gal	Gal	EY Labs

Table S3. Print list for carbohydrate microarray A and source of printed probes. All probes were printed at 1 mg/mL. Common probes for normalisation between microarrays A and B are shaded in blue below. Substitution measurement method indicated by M, MALDI; T, ToF MS; C, colourimetric assay. In house stocks synthesised as previously described [12].

Abbreviation	Probe	Source	Cat. No.	Spacer length	Structure	Substitution Average	Substitution Range
Fetuin	Fetuin from fetal bovine serum	Sigma	F2379		Glycoprotein		
ASF	Asialofetuin	Sigma			Desialylated glycoprotein		
Fibronectin	Fibronectin	Collaborative Research Inc.			Glycoprotein		
Ov	Ovalbumin from chicken egg white, grade VI	Sigma	A2512		Phosphorylated glycoprotein		
RB	RNAse B	Sigma			Glycoprotein		
Xferrin	Transferrin	Sigma			Glycoprotein		
4APHSA	4AP-HSA	In house			4AP-HSA		
α-C	α-Crystallin from bovine lens	Sigma	C4163		Phosphorylated glycoprotein		
M3BSA	Man α1,3(Man α1,6)Man-BSA	Dextra	NGP1336		Man- -(1,3)-[Man- -(1,6)-]Man-BSA	23 (T)	9 to 35
GlcNAcBSA	GlcNAc-BSA	Dextra	NGP1101	14 atom	GlcNAc-Sp14-NH2(Lys)-BSA	39 (T)	28 to 48
LacNAcBSA	LacNAc-BSA	Dextra	NGP0201		Gal- -(1,4)-GlcNAc-Sp3-BSA	11 (M)	8 to 15

3SLNB SA	3'SialylLacNAc-BSA	Dextra	NG P03 01	3 atom	Neu5Ac- -(2,3)-Gal- -(1,4)-GlcNAc-Sp3-BSA	13 (M)	8 to 25
3SLac HSA	3´-Sialyllactose-APD-HSA,	IsoSep	60/6 7		Neu5Ac- -(2,3)-Gal- -(1,4)-(Glc)-APD-HSA	6 (T)	
6SLac HSA	6´-Sialyllactose-APD-HSA,	IsoSep	60/9 3		Neu5Ac- -(2,6)-Gal- -(1,4)-(Glc)-APD-HSA	15 (T)	
2FLBS A	2'Fucosyllactose-BSA	Dextra	NG P03 07		Fuc- -(1,2)-Gal- -(1,4)-Glc-Sp3-BSA	7 (M)	4 to 10
3SFLB SA	3'Sialyl-3-fucosyllactose-BSA	Dextra	NG P04 05		Neu5Ac- -(2,3)-Gal- -(1,4)-[Fuc- -(1,3)-]Glc-Sp3-BSA	7 (M)	5 to 10
H2BS A	H Type II-APE-BSA	IsoSep	60/5 4		Fuc- -(1,2)-Gal- -(1,4)-GlcNAc- -(1-APE-BSA	26	
BGAB SA	Blood Group A-BSA	Dextra	NG P63 05	6 atom	GalNAc- -(1,3)-[Fuc- -(1,2)-]Gal-BSA	19 (M)	11 to 28
BGBH SA	Blood Group B-HSA	Dextra	NG P93 23	6 atom	Gal- -(1,3)-[Fuc- -(1,2)-]Gal-BSA	21 (M)	
Ga3GB SA	Galα1,3Gal-BSA	Dextra	NG P02 03		Gal- -(1,3)-Gal-Sp3-BSA	20 (M)	15 to 26
Gb4GB SA	Galb1,4GalBSA	Dextra	NG P02 04		Gal- -(1,4)-Gal-Sp3-BSA	16 (M)	9 to 29
Ga2GB SA	Gala1,2GalBSA	Dextra	NG P02 02		Gal- -(1,2)-Gal-Sp3-BSA	12 (M)	5 to 21

LNFPI BSA	Lacto-*N*-fucopentaose I-BSA	Dextra	NG P05 03		Fuc- -(1,2)-Gal- -(1,3)-GlcNAc- -(1,3)-Gal- -(1,4)-Glc-BSA	20 (M)	11 to 30
LNFPI IBSA	Lacto-*N*-fucopentaose II-BSA	Dextra	NG P05 01		Fuc- -(1,3)-Gal- -(1,3)-GlcNAc- -(1,3)-Gal- -(1,4)-Glc-BSA	15 (M)	6 to 31
LNFPI IIBSA	Lacto-*N*-fucopentaose III-BSA	Dextra	NG P05 02		Gal- -(1,4)-[Fuc- -(1,3)-]GlcNAc- -(1,3)-Gal- -(1,4)-Glc-BSA	20 (M)	12 to 29
LNDH IBSA	Lacto-*N*-difucohexaose I-BSA	Dextra	NG P06 01		Fuc- -(1,2)-Gal- -(1,3)-[Fuc- -(1,4)-]GlcNAc- -(1,3)-Gal- -(1,4)-(Glc)-Sp3-BSA	7.5 (T)	4 to 12
LebBS A	LNDI-BSA/ Lewis b-BSA	IsoSep	60/0 4		Fuc- -(1,2)-Gal- -(1,3)-[Fuc- -(1,4)-]GlcNAc- -(1,3)-Gal- -(1,4)-(Glc)-APD-BSA	10	
LexBS A	Lewis x-BSA	Dextra	NG P03 02		Gal- -(1,4)-[Fuc- -(1,3)-]GlcNAc-BSA	24 (M)	13 to 35
DiLex BSA	Di-Lex-APE-BSA	IsoSep	61/6 4		Gal- -(1,4)-[Fuc- -(1,3)-]GlcNAc- -(1,3)-Gal- -(1,4)-[Fuc- -(1,3)-]GlcNAc- -(1-O-APE-BSA	28 (C)	
DiLex HSA	Di-Lewisx-APE-HSA	IsoSep	61/5 9		Gal- -(1,4)-[Fuc- -(1,3)-]GlcNAc- -(1,3)-Gal- -(1,4)-[Fuc- -(1,3)-]GlcNAc- -(1-O-APE-HSA	18 (T)	
3LexH SA	Tri-Lex-APE-HSA	IsoSep	61/5 6		Gal- -(1,4)-[Fuc- -(1,3)-]GlcNAc- -(1,3)-Gal- -(1,4)-[Fuc- -(1,3)-]GlcNAc -(1,3)-Gal- -(1,4)-[Fuc- -(1,3)-]GlcNAc- -(1-O-APE-HSA	15 (T)	
3SLex BSA3	3'Sialyl Lewis x-BSA	Dextra	NG P04 03	3 atom	Neu5Ac- -(2,3)-Gal- -(1,4)-[Fuc- -(1,3)-]GlcNAc-Sp3-BSA	9 (M)	3 to 16
SLexB SA14	3'Sialyl Lewis x-BSA	Dextra	NG P14 03	14 atom	Neu5Ac- -(2,3)-Gal- -(1,4)-[Fuc- -(1,3)-]GlcNAc-Sp14-BSA	8 (T)	6 to 15

6SuLe xBSA	6-Sulfo Lewis x-BSA	Dextra	NG P06 03	(SO_4)6Gal- -(1,4)-[Fuc- -(1,3)-]GlcNAc-Sp3-BSA	8 (M)	1 to 16
6SuLea BSA	6-Sulfo Lewis a-BSA	Dextra	NG P06 04	(SO_4)6Gal- -(1,3)-[Fuc- -(1,4)-]GlcNAc-Sp3-BSA	17 (M)	8 to 28
3SuLea BSA	3-Sulfo Lewis a-BSA	Dextra	NG P03 04	(SO_4)3Gal- -(1,3)-[Fuc- -(1,4)-]GlcNAc-Sp3-BSA	15 (M)	9 to 20
PBS	PBS					
DFPL NHHS A	Difucosyl-para-lacto-N-hexaose-APD-HSA, (Lea/Lex)	IsoSep	61/5 7	Gal- -(1,3)-[Fuc- -(1,4)-]GlcNAc- -(1,3)-Gal- -(1,4)-[Fuc- -(1,3)-]GlcNAc- -(1,3)-Gal- -(1,4)-(Glc)-APD-HSA	21 (T)	
LeaBS A	Lewis a-BSA	Dextra	NG P07 04	Gal- -(1,3)-[Fuc- -(1,4)-]GlcNAc-Sp3-BSA	12 (M)	2 to 28
LeyHS A	Lewis y-tetrasaccharide-APE-HSA	IsoSep	60/9 5	Fuc- -(1,2)-Gal- -(1,4)-[Fuc- -(1,3)-]GlcNAc- -(1-O-APE-HSA	13.3 (T)	
3FLey HSA	Tri-fucosyl-Ley-heptasaccharide-APE-HSA	IsoSep	61/6 3	Fuc- -(1,2)-Gal- -(1,4)-[Fuc- -(1,3)-]GlcNAc- -(1,3)-Gal- -(1,4)-[Fuc- -(1,3)-]GlcNAc- -(1-O-APE-HSA	11 (T)	
LNnT HSA	Lacto-N-neotetraose-APD-HSA	IsoSep	60/7 2	Gal- -(1,4)-GlcNAc- -(1,3)-Gal- -(1,4)-(Glc)-APD-HSA	9.2 (M)	
LNTH SA	Lacto-N-tetraose-APD-HSA	IsoSep	60/9 7	Gal- -(1,3)-GlcNAc- -(1,3)-Gal- -(1,4)-(Glc)-APD-HSA	22 (T)	
MML NnHH SA	Monofucosyl, monosialyllacto-N-neohexaose-APD-HSA	IsoSep	61/6 2	Neu5Ac- -(2,3)-Gal- -(1,4)-GlcNAc- -(1,3)-[Gal- -(1,4)-[Fuc- -(1,3)-]GlcNAc- -(1,6)-]Gal- -(1,4)-(Glc)-APD-HSA	15 (T)	
SLNnT HSA	Sialyl-LNnT-penta-APD-HSA	IsoSep	61/6 8	Neu5Ac- -(2,3)-Gal- -(1,4)-GlcNAc- -(1,3)-Gal- -(1,4)-(Glc)-APD-HSA	9.3 (T)	

aGM1 HSA	Asialo-GM1-tetrasaccharide-APD-HSA	IsoSep	60/96		Gal- -(1,3)-GalNAc- -(1,4)-Gal- -(1,4)-(Glc)-APD-HSA	10 ©	
GlobNTHSA	Globo-N-tetraose-APD-HSA	IsoSep	60/99		GalNAc- -(1,3)-Gal- -(1,4)-Gal- -(1,4)-(Glc)-APD-HSA	8.4 (T)	
GlobT HSA	Globotriose-APD-HSA	IsoSep	60/90		Gal- -(1,4)-Gal- -(1,4)-Glc- -(1-APE-HSA	21 (T)	

Table S4. Print list for carbohydrate microarray B and source of printed probes. All probes were printed at 1 mg/mL except fibrinogen (fibrin), ovomucoid (ovomuc) and Tri-Lex-APE-HSA (3LexHSA) were printed at 0.5 mg/mL. Common probes for normalisation between microarrays A and B are shaded in blue below. Substitution measurement method indicated by M, MALDI; T, ToF MS; C, colourimetric assay. In house stocks synthesised as previously described [12].

Abbreviation	Neoglycoconjugate	Source	Cat. No.	Spacer Length	Structure	Substitution Average	Substitution Range
Fetuin	Fetuin from fetal bovine serum	Sigma	F2379		Glycoprotein		
Inv	Invertase, grade VII	Sigma	I4504		Glycoprotein		
Fibrin	Fibrinogen	Sigma	F-4129		Glycoprotein		
Ov	Ovalbumin from chicken egg white, grade VI	Sigma	A2512		Phosphorylated glycoprotein		
PBS	PBS						
A1AT	alpha-1-antitrypsin	Sigma	A-6388		Glycoprotein		
4APHSA	4AP-HSA	In house			4AP-HSA		
α -C	α-Crystallin from bovine lens	Sigma	C4163		Phosphorylated glycoprotein		
M3BSA	Manα1,3(Manα1,6)Man-BSA	Dextra	NGP1336		Man- -(1,3)-[Man- -(1,6)-]Man-BSA	23 (T)	9 to 35
GlcNAcBSA	GlcNAc-BSA	Dextra	NGP1101	14 atom	GlcNAc-Sp14-NH2(Lys)-BSA	39 (T)	28 to 48
Cerulo	Ceruloplasmin, human, type III	Sigma	C-3007		Glycoprotein	11 (M)	8 to 15

AGP	alpha-1-acid glycoprotein, human	Sigma	G9885		Glycoprotein	13 (M)	8 to 25
3SLacHSA	3´-Sialyllactose-APD-HSA	IsoSep	60/67		Neu5Ac- -(2,3)-Gal- -(1,4)-(Glc)-APD-HSA	6 (T)	
6SLacHSA	6´-Sialyllactose-APD-HSA	IsoSep	60/93		Neu5Ac- -(2,6)-Gal- -(1,4)-(Glc)-APD-HSA	15 (T)	
LacNAcaBSA	LacNAc-α-4AP-BSA	In house			LacNAc-α-4AP-BSA		
LacNAcb4AP BSA	LacNAc-β-4AP-BSA	In house			LacNAc-β-4AP-BSA		
H2BSA	H Type II-APE-BSA	IsoSep	60/54		Fuc- -(1,2)-Gal- -(1,4)-GlcNAc- -(1-APE-BSA	26	
PBS	PBS						
Ovomuc	Ovomucoid	Dextra	T-9253		Glycoprotein		
Ga3GBSA	Galα1,3Gal-BSA	Dextra	NGP0203	3 at om	Gal- -(1,3)-Gal-Sp3-BSA	20 (M)	15 to 26
RhaBSA	L-Rhamnose-BSA	Dextra	NGP1106	14 atom	L-Rhamnose-Sp14-BSA	32 (M)	16 to 54
PBS	PBS						
LNFPIBSA	Lacto-*N*-fucopentaose I-BSA	Dextra	NGP0503		Fuc- -(1,2)-Gal- -(1,3)-GlcNAc- -(1,3)-Gal- -(1,4)-Glc-BSA	20 (M)	11 to 30
XManaBSA	Man-α-ITC-BSA	In house			Man-α-ITC-BSA		
PBS	PBS	Dextra					
XManbBSA	Man-β-4AP-BSA	In house			Man-β-4AP-BSA		
LebBSA	LNDI-BSA/ Lewis b-BSA	IsoSep	60/04		Fuc- -(1,2)-Gal- -(1,3)-[Fuc- -(1,4)-]GlcNAc- -(1,3)-Gal- -(1,4)-(Glc)-APD-BSA	10	
LexBSA	Lewis x-BSA	Dextra	NGP0302		Gal- -(1,4)-[Fuc- -(1,3)-]GlcNAc-BSA	24 (M)	13 to 35
XGalbBSA	Gal-β-ITC-BSA	In house					
XylbBSA	Xyl-β-4AP-BSA	In house					
3LexHSA	Tri-Lex-APE-HSA	IsoSep	61/56		Gal- -(1,4)-[Fuc- -(1,3)-]GlcNAc- -(1,3)-Gal- -(1,4)-[Fuc- -(1,3)-]GlcNAc -(1,3)-Gal- -(1,4)-[Fuc- -(1,3)-]GlcNAc- -(1-O-APE-HSA	15 (T)	
XylaBSA	Xyl-α-4AP-BSA	In house			Xyl-α-4AP-BSA		

XGlcbBSA	Glc-β-4AP-BSA	In house			Glc-β-4AP-BSA		
FucaBSA	Fuc-α-4AP-BSA	In house			Fuc-α-4AP-BSA		
6SuLeaBSA	6-Sulfo Lewis a-BSA	Dextra	NGP0604	3 atom	(SO_4)6Gal- -(1,3)-[Fuc- -(1,4)-]GlcNAc-Sp3-BSA	17 (M)	8 to 28
FucbBSA	Fuc-β-4AP-BSA	In house			Fuc-β-4AP-BSA		
GlcbITCBSA	Glc-β-ITC-BSA	In house			Glc-β-ITC-BSA		
Galb4APBSA	Gal-β-4AP-BSA	In house			Gal-β-4AP-BSA		
Neu5GcBSA	Neu5Gc-BSA	In house			Neu5Gc-BSA		
LeyHSA	Lewis y-tetrasaccharide-APE-HSA	IsoSep	60/95		Fuc- -(1,2)-Gal- -(1,4)-[Fuc- -(1,3)-]GlcNAc- -(1-O-APE-HSA	13.3 (T)	
PBS							
PBS							
LNTHSA	Lacto-N-tetraose-APD-HSA	IsoSep	60/97		Gal- -(1,3)-GlcNAc- -(1,3)-Gal- -(1,4)-(Glc)-APD-HSA	22 (T)	
PBS							
PBS							
D-GlobTHSA	Globotriose-HSA	Dextra	NGP2340	3 atom	Gal- -(1,4)-Gal- -(1,4)-Glc-Sp3-BSA	12.7 (M)	8 to 19
GlobNTHSA	Globo-N-tetraose-APD-HSA	IsoSep	60/99		GalNAc- -(1,3)-Gal- -(1,4)-Gal- -(1,4)-(Glc)-APD-HSA	8.4 (T)	
GlobTHSA	Globotriose-APE-HSA	IsoSep	60/90		Gal- -(1,4)-Gal- -(1,4)-Glc- -(1-APE-HSA	21 (T)	

Materials and Methods

Assay for Retained PNAG after Washing.

Cultures were grown overnight in BHI NaCl, washed three times in TBS and cells were adjusted to an absorbance of approximately 1.0 at 595 nm. Bacteria cultures were placed in to tubes in 1 mL aliquots to act as a positive control and were set aside. Separate 1 mL cultures were washed one to five times by resuspending in TBS, centrifuging the bacteria in to a pellet at 5,000 x g and removing the supernatant each time. After the final wash, bacterial pellets were resuspended in 1 mL TBS or TBS with 0.05%, 0.02% or 0.01% (v/v) Tween® 20. Washed and unwashed 1 mL bacterial suspensions were collected by centrifugation (5,000 × g for 5 min), resuspended in 250 µL of 0.5 M ethylenediaminetetraacetic acid (EDTA) and boiled for 5 min. Samples were centrifuged and 40 µL aliquots of the supernatant were treated with proteinase K (10 µL of 20 µg/mL) at 65 °C for 1 h and then boiled again for 5 min. The proteinase K-treated samples (2 µL) were pipetted on to a PVDF membrane in triplicate and the membrane was blocked and probed for PNAG using anti-PNAG IgG1 mAb as described above. After imaging HRP activity on the membrane, digital images (.jpg) were saved and used to relatively quantify the amounts of PNAG present on the membrane compared to the control (PNAG without three washes) using ImageJ software (National Institutes of Health, Bethesda, MD, U.S.A.). Measurements using the same size frame were taken for each spot, the frames were analysed and the resulting data was exported into Excel v.2010 (Microsoft). The mean of technical triplicates was taken for each condition and expressed as a percentage of intensity of unwashed cells.

References

1. Horsburgh, M.J.; Aish, J.L.; White, I.J.; Shaw, L.; Lithgow, J.K.; Foster, S.J. Σ^b modulates virulence determinant expression and stress resistance: Characterization of a functional *rsbu* strain derived from *Staphylococcus aureus* 8325-4. *J. Bacteriol.* **2002**, *184*, 5457–5467.
2. McKenney, D.; Pouliot, K.L.; Wang, Y.; Murthy, V.; Ulrich, M.; Döring, G.; Lee, J.C.; Goldmann, D.A.; Pier, G.B. Broadly protective vaccine for *Staphylococcus aureus* based on an *in vivo*-expressed antigen. *Science* **1999**, *284*, 1523–1527.
3. Jefferson, K.K.; Cramton, S.E.; Götz, F.; Pier, G.B. Identification of a 5-nucleotide sequence that controls expression of the ica locus in *staphylococcus aureus* and characterization of the DNA-binding properties of icar. *Mol. Microbiol.* **2003**, *48*, 889–899.
4. Fitzpatrick, F.; Humphreys, H.; O'Gara, J.P. Evidence for *icaadbc*-independent biofilm development mechanism in methicillin-resistant *Staphylococcus aureus* clinical isolates. *J. Clin. Microbiol.* **2005**, *43*, 1973-1976.
5. Houston, P.; Rowe, S.E.; Pozzi, C.; Waters, E.M.; O'Gara, J.P. Essential role for the major autolysin in the fibronectin-binding protein-mediated *Staphylococcus aureus* biofilm phenotype. *Infect. Immun.* **2011**, *79*, 1153–1165.
6. Choi, A.H.K.; Slamti, L.; Avci, F.Y.; Pier, G.B.; Maira-Litrán, T. The *pgaabcd* locus of *Acinetobacter baumannii* encodes the production of poly-β-1-6-*N*-acetylglucosamine, which is critical for biofilm formation. *J. Bacteriol.* **2009**, *191*, 5953–5963.
7. O'Neill, E.; Pozzi, C.; Houston, P.; Smyth, D.; Humphreys, H.; Robinson, D.A.; O'Gara, J.P. Association between methicillin susceptibility and biofilm regulation in *Staphylococcus aureus* isolates from device-related infections. *J. Clin. Microbiol.* **2007**, *45*, 1379–1388.
8. O'Neill, E.; Pozzi, C.; Houston, P.; Humphreys, H.; Robinson, D.A.; Loughman, A.; Foster, T.J.; O'Gara, J.P. A novel *Staphylococcus aureus* biofilm phenotype mediated by the fibronectin-binding proteins, Fnbpa and Fnbpb. *J. Bacteriol.* **2008**, *190*, 3835–3850.
9. Kennedy, C.A.; O' Gara, J.P. Contribution of culture media and chemical properties of polystyrene tissue culture plates to biofilm development by *Staphylococcus aureus*. *J. Med. Microbiol.* **2004**, *53*, 1171–1173.

10. Kilcoyne, M.; Twomey, M.E.; Gerlach, J.Q.; Kane, M.; Moran, A.P.; Joshi, L. *Campylobacter jejuni* strain discrimination and temperature-dependent glycome expression profiling by lectin microarray. *Carbohydr. Res.* **2014**, *389*, 123–133.
11. Maira-Litrán, T.; Kropec, A.; Abeygunawardana, C.; Joyce, J.; Mark, G.; Goldmann, D.A.; Pier, G.B. Immunochemical properties of the staphylococcal poly-*N*-acetylglucosamine surface polysaccharide. *Infect. Immun.* **2002**, *70*, 4433–4440.
12. Kilcoyne, M.; Gerlach, J.Q.; Kane, M.; Joshi, L. Surface chemistry and linker effects on lectin–carbohydrate recognition for glycan microarrays. *Anal. Methods* **2012**, *4*, 2721–2728.
